# The Association of Metabolomic Profiles of a Healthy Lifestyle with Heart Failure Risk in a Prospective Study

**DOI:** 10.3390/nu15132934

**Published:** 2023-06-28

**Authors:** Yuanyuan Ma, Maomao Chu, Zuqiang Fu, Qian Liu, Jingjia Liang, Jin Xu, Zhenkun Weng, Xiu Chen, Cheng Xu, Aihua Gu

**Affiliations:** 1 State Key Laboratory of Reproductive Medicine and Offspring Health, School of Public Health, Nanjing Medical University, Nanjing 211166, China; 2 Collaborative Innovation Center for Cardiovascular Disease Translational Medicine, Nanjing Medical University, Nanjing 211166, China; 3 Department of Toxicology, Center for Global Health, Nanjing Medical University, Nanjing 211166, China; 4 School of Biomedical Engineering and Informatics, Nanjing Medical University, Nanjing 211166, China; 5 School of Public Health, Southeast University, Nanjing 211189, China; 6 Department of Maternal, Child, and Adolescent Health, School of Public Health, Nanjing Medical University, Nanjing 211166, China

**Keywords:** healthy lifestyle, metabolomics profiling, heart failure, UK biobank

## Abstract

Lifestyle has been linked to the incidence of heart failure, but the underlying biological mechanisms remain unclear. Using the metabolomic, lifestyle, and heart failure data of the UK Biobank, we identified and validated healthy lifestyle-related metabolites in a matched case-control and cohort study, respectively. We then evaluated the association of healthy lifestyle-related metabolites with heart failure (HF) risk and the added predictivity of these healthy lifestyle-associated metabolites for HF. Of 161 metabolites, 8 were identified to be significantly related to healthy lifestyle. Notably, omega-3 fatty acids and docosahexaenoic acid (DHA) positively associated with a healthy lifestyle score (HLS) and exhibited a negative association with heart failure risk. Conversely, creatinine negatively associated with a HLS, but was positively correlated with the risk of HF. Adding these three metabolites to the classical risk factor prediction model, the prediction accuracy of heart failure incidence can be improved as assessed by the C-statistic (increasing from 0.806 [95% CI, 0.796–0.816] to 0.844 [95% CI, 0.834–0.854], *p*-value < 0.001). A healthy lifestyle is associated with significant metabolic alterations, among which metabolites related to healthy lifestyle may be critical for the relationship between healthy lifestyle and HF. Healthy lifestyle-related metabolites might enhance HF prediction, but additional validation studies are necessary.

## 1. Introduction

Heart failure (HF) is a syndrome in which the heart is unable to meet the metabolic need for activity or is accompanied by elevated intracardiac pressures due to functional or structural impairment [1,2,3]. According to available data, about 64.3 million people worldwide suffered from heart failure in 2017 [4]. HF seriously endangers human health with a high admission rate, high mortality rate, and poor prognosis. In addition, its 5-year mortality rate can reach 45–60% [5]. AHA/CAC/HFSA Guideline 2022 states that a healthy lifestyle, including physical activity, maintaining a normal weight, maintaining a healthy diet, and quitting smoking, is essential to reduce the risk of HF [6]. Previous studies have demonstrated that maintaining four healthy lifestyle factors (not smoking, BMI, physical activity, and diet) can reduce the risk of HF by 45% to 81% [7,8,9], and this protective effect was found to be more pronounced in women [10]. However, there is little research on the mechanisms of a healthy lifestyle improving the incidence of heart failure.

Recently, some studies have found that metabolites were associated with the incidence of heart failure through metabolic profiling [11]. Metabolomics is a measurement in biological systems that qualitatively or quantitatively analyses all low-molecular-weight metabolites in a specific physiological period, employing high-throughput detection and data processing [12,13]. The most used metabolic profiling methods were nuclear magnetic resonance (NMR) and mass spectrometry (MS). NMR is a non-targeted metabolic profiling method that can detect any molecules containing carbon or hydrogen, while MS cannot be used to detect lipoproteins [14]. In the treatment of body fluids, NMR usually does not require chemical treatment of samples [15]. Therefore, NMR provides reproducible and low-cost high-throughput metabolite qualification. Existing studies have found that metabolites related to coffee intake are associated with the risk of diabetes through serum metabolomics, and these metabolites can add the predictivity of the risk of diabetes [16]. The blood metabolomics of HF patients are different from those of healthy individuals [17]. Moreover, smoking, as an important risk factor for heart failure, can cause changes in a variety of metabolites in NMR metabolomics profiling [14]. However, there are few epidemiological studies on the association of metabolomics with healthy lifestyles and heart failure.

Using the latest research data from the UK Biobank study, we construct the healthy lifestyle score (HLS) based on the lifestyle data to identify and verify the lifestyle-related metabolites in the discovery and validation set. Then, we validated the relation between the selected healthy lifestyle-related metabolites and HF risk. We also examined whether adding these selected metabolites to the designed model can improve the prediction of heart failure risk and conducted a sensitivity analysis to confirm the results of the prediction model.

## 2. Materials and Methods

### 2.1. Study Design and Population

The UK Biobank study is a large cohort study based on population, and details of its study design and population have been described previously [18]. In summary, the study of the UK Biobank began on 13 March 2006 and continued until 1 October 2010. More than half a million participants aged between 40 and 69 were recruited by mail at 22 United Kingdom, Scotland, and Wales assessment centres. Informed consent was obtained from each participant, and lifestyle-related information was gathered through questionnaires, interviews, and physical measurements. As well as collecting non-fasting venous blood, the last food or drink consumed was noted. Samples should be treated and stored at −80 °C after minimal treatment at the evaluation centre. The UK Biobank has received ethical approval from the UK Biobank Research Ethics Committee and Human Tissue Authority.

**Figure 1:**
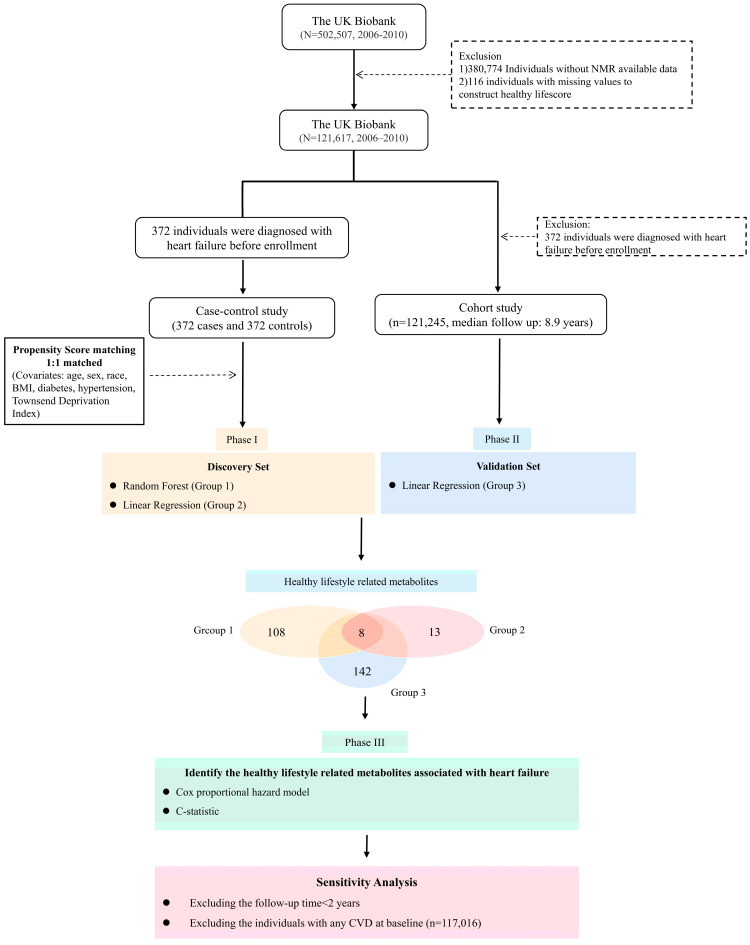
Flowchart of study participant selection and analysis process. Abbreviation: NMR, nuclear magnetic resonance; BMI, body mass index; CVD, cardiovascular disease.

This study was conducted in three phases: (a) First, the HF patients before enrolment were selected, and we performed propensity score matching to match 1:1 healthy control subject, according to covariates including age, sex, race, BMI, diabetes at baseline, hypertension, and Townsend deprivation index (372 control subjects, 372 cases). We utilised nonlinear methods (random forest algorithm) and linear regression to identify metabolites related to HLS. (b) A random subset of 121,733 (24%) of the original 502,507 UK Biobank participants had NMR biomarker data. Study participants with previous heart failure were excluded, and a subsequent cohort study was conducted to further examine the selected healthy lifestyle-related metabolites using linear regression. (c) Finally, we calculated the risk ratio (HRs) and 95% CI of healthy lifestyle-related metabolites associated with the risk of heart failure through Cox proportional hazards regression. To evaluate the predictability of metabolites related to healthy lifestyles to the risk of heart failure, we calculated the *C*-statistics and integrated discrimination improvement (IDI). Sensitivity analyses were performed to confirm the association between heart failure and healthy lifestyle-related metabolites. A flowchart of the study process is shown in Figure 1.

### 2.2. Healthy Lifestyle Score (HLS)

We used four lifestyle factors (smoking, drinking, physical activity, and diet) to construct the HLS, which coincided with the recommendations from the AHA/ACC/HFSA Guideline [19,20,21]. The definitions of a healthy level in lifestyle factors are as follows: (a) Smoking status was classified as “current”, “past”, or “never”. Never smoking is considered a health standard. (b) The frequency and amount of alcohol consumed are currently self-reported, and health levels are defined as one or fewer drinks per day for women and two or fewer drinks per day for men (14 g per glass in the US and 8 g per glass in the UK) [20,22]. (c) With regard to physical activity, “regular physical activity” refers to a person who performs at least 75 min of strenuous activity or 150 min of moderate physical activity (or equivalent combination) every week or performs strenuous activity at least once a week and performs a moderate physical activity for 5 days [23]. (d) “Healthy diet” refers to a diet pattern that met at least five items of the recommendations: abundant consumption of fruit, vegetables, whole grains, fish, shellfish, dairy products, and vegetable oils, and a decreased overall consumption of refined grains, processed meats, as well as unprocessed meats, and sugar-sweetened beverages [21]. Detailed criteria and scores for health levels are provided in Table S1. The HLS ranges from 0 to 4. The higher the index, the healthier the lifestyle.

### 2.3. Metabolomics Profiling

Approximately 118,000 venous blood samples were collected from baseline recruitment and stored at −80 °C. Metabolomics analysis of plasma samples were performed using the NMR high-throughput platform in Nightingale Health’s laboratories of Finland. Details of the metabolic profiling platform and experimentation have been described previously [24,25] and can be found in UK Biobank online resources [26]. A total of 249 metabolic biomarkers, including low molecular metabolic biomarkers, such as lipids, fatty acids, amino acids, and ketones, as well as the distribution, particle size, and composition of lipoprotein subclasses, were quantified per plasma sample. The coefficient of variation (CV) was below 5% for the majority of the metabolites. The unnamed metabolites and metabolites with no between-person variations were removed. Each missing data point was estimated with half of the minimum measured value. Finally, a total of 161 metabolites were included in the analysis (Table S2) and the matrix analysis of all metabolic molecules was performed (Figure S1).

### 2.4. Outcomes

Heart failure was defined by self-report at baseline or the records of initial diagnosis and major causes of death from hospitals in England, Wales, and Scotland. Cases of heart failure were coded according to the International Classification of Diseases (ICD-10), and the disease code involved in heart failure included I11, I13, and I50.

### 2.5. Covariates

The covariates were considered in the existing study as follows: demographic characteristics, including age (years), sex (male/female), race (white or other), and Townsend deprivation index (continuous); anthropometric measures, including body mass index (BMI) (<25 kg/m^2^, 25 to 29.9 kg/m^2^, ≥30 kg/m^2^); lifestyle-related factors, including smoking status (current/past/never), alcohol drinking status (current/past/never), and physical activity (continuous); and underlying disease, including diabetes, hypertension, and family history of cardiovascular disease (CVD) (yes/no). Detailed information was obtained from the baseline interview questionnaire or physical measurements, and the disease status was obtained through self-report. The Townsend deprivation index is a measure of socioeconomic status based on the economic output of the participant’s region. Physical activity is measured as total metabolic equivalent (min/week), including walking and all other activities of moderate or vigorous intensity.

### 2.6. Statistical Analyses

In this study, baseline characteristics of the study population were stratified by heart failure. The age distribution was reported as the mean ± standard deviation (SD), while the distribution of healthy lifestyle factors and related covariates were presented as percentages (%). Comparisons of baseline characteristics across the categories of the overall heart failure were made using chi-squared tests. 

The NMR biomarker data was log10 transformed since it did not meet the normal distribution. In phase I analysis, we used nonlinear methods (random forest algorithm) and linear regression to preliminary screen for metabolites associated with a healthy lifestyle score among 161 metabolites in a matched case-control population. Because of the collinearity of our study factor HLS with smoking, alcohol consumption, and physical activity levels, adjustments for such covariates were excluded from the linear regression analyses of the healthy lifestyle score with metabolites. In phase II analysis, we examined the association of metabolite with a healthy lifestyle by linear regression after adjusting for potential confounders, mainly including the basic classical factors (age, sex, race, BMI, diabetes, hypertension, Townsend deprivation index, and family history of CVD). In phase III analysis, we used Cox regression to calculate hazard ratios (HRs) and 95% confidence intervals (CIs) between healthy lifestyle-related metabolites and the risk of heart failure after adjustment for covariates. To evaluate the predictivity of healthy lifestyle-related metabolites for heart failure, we calculated the *C*-statistic and performed the receiver operating characteristic (ROC) analysis. We considered three models: (1) a model using traditional risk factors such as age, race, BMI, smoking status, alcohol status, physical activity, Townsend Deprivation Index, diabetes, and hypertension; (2) a model based on healthy lifestyle-related metabolites that was associated with heart failure risk in the current studies; (3) a model based on a combination of traditional risk factors and metabolites. We compared *C*-statistics among these models using nonparametric methods.

The data processing and analyses of baseline characteristics were conducted by STATA MP16 (SAS Institute, Cary, NC, USA), and analyses in the above three phases were performed using R 4.1.3 (R-Foundation for Statistical Computing, Vienna, Austria). All statistical tests were two sided. We used Bonferroni correction to correct for multiple testing at the α = 0.05 significance level.

## 3. Results

### 3.1. Baseline Characteristics

Table 1 shows the baseline characteristics of the cohort population according to the outcome of heart failure. The mean age (SD) of the cohort was 56.5 (SD) years, and 45.8% were male. During a follow-up of approximately 1.06 million person-years (median 8.9 years [IQR 8.2–9.5]), 1718 cases of heart failure were identified. Men accounted for a larger proportion of heart failure cases, and smoking, alcohol consumption, and obesity were more common among cases. In the 1:1 matched case-control population, the distribution of factors such as age, sex, healthy lifestyle, and BMI was basically the same in the cases and controls, and the bias of each factor was less than 10% between the two groups (Table S3).

### 3.2. Identification of Healthy Lifestyle-Related Metabolites

In phase I analysis, 108 metabolites associated with a healthy lifestyle were screened by a random forest algorithm (Figure S2) from a matched case-control population. Moreover, 13 healthy lifestyle-related metabolites were validated through linear regression (*p*-value < 0.05) (Table S4). We performed principal component analysis (PCA) of these metabolites to reveal the clustering patterns of each metabolite (Figures S3–S5). Figure S4 shows the contribution rate of each principal component to the total variation. There were eight metabolites overlapping in the two-stage screening of random forest and linear regression, including docosahexaenoic acid, omega-3 fatty acids, polyunsaturated fatty acids to total fatty acids percentage, monounsaturated fatty acids to total fatty acids percentage, linoleic acid to total fatty acids percentage, omega-6 fatty acids to total fatty acids percentage, creatinine, and glycoprotein acetyls.

In phase II analysis, the 161 metabolites included in existing research were validated in the cohort study. After adjusting for potential confounders and considering multiple tests, 142 healthy lifestyle-related metabolites were identified using linear regression (Bonferroni *p*-value < 3.11 × 10^−4^), in which 119 metabolites were negatively correlated with the healthy lifestyle score, and 23 metabolites were positively correlated. See Table S5 for detailed results. The eight healthy lifestyle-related metabolites selected from phase I were all verified in the cohort population. In addition, linear regression was used to examine the associations between the eight metabolites and the individual components of the healthy lifestyle score. The results showed that the above eight metabolites were associated with the four lifestyle factors included in the study. The detailed results are shown in Table S6.

### 3.3. Association of Healthy Lifestyle-Related Metabolites with Heart Failure

**Figure 2:**
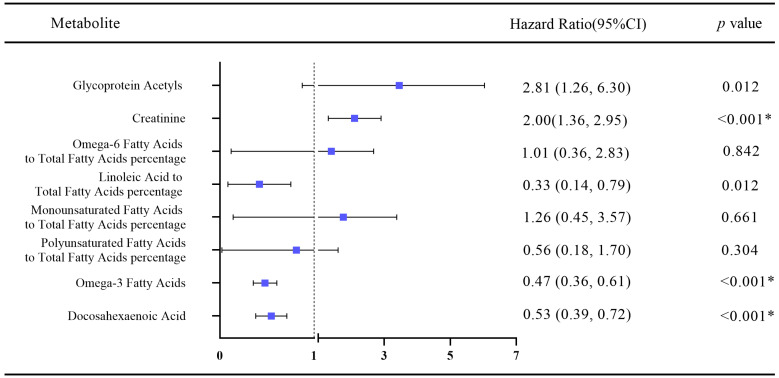
Hazard ratio of healthy lifestyle-related metabolites associated with heart failure risk. Of the above eight metabolite indicators, omega-3 fatty acids and docosahexaenoic acid (DHA) showed inverse associations with heart failure risk, whereas the creatinine showed positive associations with heart failure risk. HR, hazard ratio. * Significance, *p* < 0.001.

After adjustment for multiple variables (age, sex, race, BMI, physical activity, smoking, drinking, diabetes, hypertension, Townsend deprivation index, and family history of CVD), we calculated the hazard ratio between the healthy lifestyle score and heart failure through Cox regression. Three of the eight healthy lifestyle-related metabolites were associated with heart failure risk (Bonferroni *p*-value < 3.11 × 10^−4^). As shown in Figure 2, omega-3 fatty acids and docosahexaenoic acids, which were positively correlated with healthy living scores, were associated with a low risk of heart failure, with a hazard ratio (HR) of 0.47 ([95% CI 0.36, 0.61], *p*-value < 0.001) and 0.53 ([95% CI 0.39, 0.72], *p*-value < 0.001), respectively. Creatinine, a metabolite negatively correlated with healthy lifestyle score, was associated with a high risk of heart failure, with HR values of 2.00 ([95% CI 1.32, 2.95], *p*-value < 0.001).

**Figure 3:**
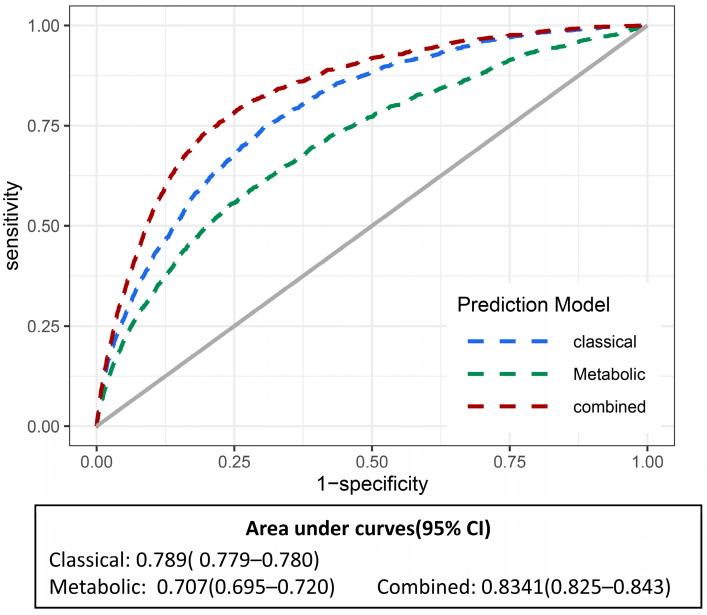
Receiver operating characteristic curves (ROC) of different prediction models in cohort study. The classical factors included age, sex, race, BMI, smoking status, alcohol status, physical activity, diabetes, hypertension, and Townsend deprivation index, and the combined model involved healthy lifestyle-related metabolites associated with heart failure risk in the metabolic model. *p* < 0.001 for the combined model vs. classical factor model, and *p* < 0.001 for metabolic model vs. classical factor model. The gray line is for reference.

For the predictive analysis of heart failure (Figure 3), we used three metabolites related to a healthy lifestyle and used classical risk factors to obtain the *C*-statistics of the model. The *C*-statistic increased from 0.806 [95% CI, 0.796–0.816] to 0.844 [95% CI, 0.834–0.854] (*p*-value < 0.001) when metabolites were added to the classical risk factor model. The IDI was 0.002 [95% CI, 0.001–0.004] (*p*-value < 0.001), and the NRI was 0.048 [95% CI, 0.011–0.077] (*p*-value = 0.02) (Table S7). The model combining classical factors and metabolites significantly improved the predictivity of heart failure. Subsequently, we performed sensitivity analysis, excluding individuals in the cohort who were followed up for less than 2 years and who had CVD at the time of enrolment (Table S8), and the *C*-statistic results also showed that the model combining metabolites had a higher predictive ability for heart failure.

## 4. Discussion

In this study, we identified and verified eight metabolite indicators related to a healthy lifestyle based on NMR metabolomics data from the UK Biobank. We found that omega-3 fatty acids and DHA, which were positively correlated with a healthy lifestyle, showed inverse associations with heart failure risk. However, the creatinine, which was negatively associated with a healthy lifestyle, showed positive associations with heart failure risk. The three metabolites above can be used as potential biomarkers of heart failure and significantly improve the predictivity of classical risk factor models for heart failure. The findings of the current study provide new insights into the mechanisms, by which a healthy lifestyle reduces heart failure risk and demonstrate the ability of healthy lifestyle-related metabolites to improve the prediction of heart failure.

Numerous studies have shown that different exposure factors, such as individual lifestyle, anthropometric parameters, and diet, are associated with various metabolic signatures. In this study, we found that the healthy lifestyle score combined with smoking, alcohol, diet, and physical activity level was associated with changes in lipid metabolism, mainly including very low-density lipoprotein (VLDL) and high-density lipoprotein (HDL), which was consistent with a study from the China Kadoorie Biobank [27]. In addition, our results also demonstrated that a healthy lifestyle caused changes in creatinine, albumin, and some amino acid metabolites, which is consistent with the results of healthy lifestyle-induced changes in plasma metabolism in a Spanish cohort study [28]. These changes in lipid metabolism and serum metabolic profile may be associated with the development of coronary heart disease and type 2 diabetes [27,28]. Other studies have found that a healthy lifestyle can also cause changes in the serum metabolism (including hexose, glutamate, sphingolipids, and phospholipids) [29] and urine metabolic profile (including trimethylamine *N*-oxide (TMAO) and dimethylamine) [30] of different metabolites. More recent studies have used metabolite phenotypes, identifying metabolite alterations in healthy diet patterns, to objectively assess dietary patterns. The healthy diet indicator is related to serum metabolites, including polyunsaturated fat and fibre components, which is similar to the results of this study [31]. In addition, several studies have shown that lifestyle factors such as BMI [32], alcohol intake [33], and physical activity [34] can cause changes in amino acids, fatty acids, lipoproteins, and body fluid balance metabolites. These results may explain the protective effect of a healthy lifestyle on metabolic diseases from the perspective of metabolomics.

Metabolomics provides a new insight into risk factors and CVD development. There has been research based on the Framingham Heart Study showing that glycerolipid metabolism mediated the association of the cardiovascular health (CVH) score with HF [35]. To our knowledge, we are the first to explain the association of the incident of heart failure with healthy lifestyle-related metabolic profiling. We observed that a healthy lifestyle can cause changes in metabolites, and the changes were related to the risk of heart failure. In the current study, omega-3 fatty acids and DHA, which were positively correlated with HLS, were inversely associated with the risk of heart failure. Thus, these metabolites can be considered protective factors for heart failure (HR: 0.47 [95% CI 0.36, 0.61]; 0.53, [95% CI 0.39, 0.72]). The omega-3 family of polyunsaturated fatty acids includes a variety of fatty acids [36]. The types of fatty acid metabolites included in this study were omega-3 fatty acids, DHA, omega-6 fatty acids, linoleic acid, and the total amount of polyunsaturated fatty acids, monounsaturated fatty acids, and saturated fatty acids. Only omega-3 fatty acids and DHA were found to be inversely associated with the risk of heart failure. Fish are the main source of omega-3 fatty acids in the human body [37], and the intake of omega-3 fatty acids and fish can reduce the risk of heart failure [38]. The definition of a healthy diet in this study included the criteria for fish intake, and there were no detailed criteria for specific food sources of other essential fatty acids, so we speculate that this may be the reason why only omega-3 and DHA were found to have an inverse association with heart failure in our results. A cohort study revealed that patients with compensatory heart failure had reduced levels of omega-3 fatty acids [39]. When the intake of omega-3 fatty acids reaches 1 g per day (850–882 mg eicosapentaenoic acid and docosahexaenoic acid as ethyl esters in the average ratio of 1:1.2), the mortality and admission rate of patients can be reduced in those with chronic heart failure and already on recommended therapy [40]. Meanwhile, studies have shown that omega-3 fatty acid supplementation reduces the incidence of sudden cardiac death and improves left ventricular ejection fraction (LVEF%), thereby reducing the incidence of heart failure, but does not significantly improve the mortality of heart failure [41]. Unlike omega-3 fatty acids, DHA is associated with the prognosis of heart failure, and high plasma DHA content reduces all-cause mortality in patients with acute decompensated heart failure with preserved ejection fraction (HfpEF) [42]. These findings suggest that omega-3 fatty acids and DHA may play a role in preventing heart failure and improving the outcome of heart failure, which is consistent with our findings. However, recent studies indicate that the impact of omega-3 supplementation on heart health is multifaceted and has the potential to raise the risk of atrial fibrillation (AF). Among patients opting for a high dosage of 4 g/day of omega-3 fatty acids, the hazard ratio for AF was 1.69 [95% CI (1.29, 2.21)] [43]. However, no noticeable elevation in AF risk was observed with either an intermediate dosage of 1.8 g/day or a standard daily dose of 840 mg/day [44,45]. Additional research is necessary to validate the cardioprotective impact of omega-3 fatty acid supplementation. In addition, our results also found that creatinine, which was negatively correlated with healthy lifestyle scores, was positively associated with the risk of heart failure, namely, it was a risk factor for heart failure (HR: 2.00, [95% CI 1.36, 2.95]). Serum creatinine levels can be affected by diet, physical activity, and other factors [46]. Elevated serum creatinine levels commonly indicate acute deterioration of renal function, thus affecting the prognosis of patients with heart failure [47]. However, it has been found that the prediction based on serum creatinine and cystatin C is not accurate enough to detect the actual change in glomerular filtration rate (GFR) in hospitalised patients with acute heart failure [48], so the prediction of creatinine for the prognosis of heart failure needs further research. The findings of our study revealed the healthy lifestyle-related metabolomic signatures in heart failure, suggesting a new insight to heart failure diagnosis and prevention.

Several potential mechanisms might underlie the observed relations between a healthy lifestyle and HF. Animal studies have shown that exercise training (ExT) can reverse cardiac aging phenotypes associated with heart failure with preserved ejection fraction (HfpEF) in male mice, and its molecular mechanism involves that ExT reversing the downregulation of fatty acid metabolism pathways related to aging and ultimately regulating the cell cycle and cell division [49]. Other animal studies in mice have also shown that the improvement of heart failure by exercise is related to the recovery of oxidative metabolism [50], the β3-AR-nNOS-NO pathway [51], autonomic imbalance, and impaired calcium homeostasis [52]. A high-fibre diet and acetate supplementation can prevent the development of heart failure, and the mechanism may be related to changes in gut microbiota and can reduce the expression of the cardiovascular regulator Egr1, thereby improving the occurrence of cardiac hypertrophy, fibrosis, and inflammation [53]. Furthermore, dietary supplementation with *n*-3 polyunsaturated fatty acids (PUFAs) can inhibit the cardiac fibrosis in HF by the interaction with nuclear factor erythropoietin 2 related factor 2 (Nrf2), G-protein coupled receptor (GPR), or free fatty acid receptor 4 (Ffar4) [54,55]. In addition, studies have shown that heart biopsies from patients with heart failure show an accumulation of glucose in the heart, and a ketogenic diet (a high-fat, low-carbohydrate, low-protein diet) can prevent the occurrence of heart failure in diabetic mice. The results may be related to the reduced expression of pyruvate carriers during heart failure, which leads to reduced glucose utilisation and accumulation in the heart [56]. Smoking is a risk factor for heart failure, which may increase the risk of heart failure through the NO pathway affecting lipid metabolism, and immune and inflammatory responses [57,58,59]. However, the conclusions of different studies about the relation of alcohol and heart failure were not consistent according to alcohol consumption. Studies have shown that excessive alcohol consumption can lead to myocardial cell dysfunction (abnormal calcium homeostasis), increased norepinephrine levels, and decreased myocardial contractility, leading to alcoholic cardiomyopathy [60,61,62]; moderate alcohol consumption has been shown to have a certain preventive effect on the prevention of heart failure, which may be related to the increase in high-density lipoprotein cholesterol, insulin sensitivity, plasma adiponectin level, inhibition of inflammation, and improvement of endothelial function.

The strengths of this study can be attributed to the large sample size, with many patients diagnosed with heart failure in a cohort population. Moreover, we obtained detailed information about the lifestyle and diet of the population and were able to adjust for potential confounders, construct a healthy lifestyle score, and observe the effect of healthy lifestyle on metabolic changes and its association with heart failure. Another strength is to use NMR metabolomics to observe the changes in various biochemical substances, and to identify the intermediates between healthy lifestyle and the pathogenesis of heart failure. Additionally, we internally validated the association of healthy lifestyle-related metabolites with heart failure using samples from the same cohort and the same metabolomic profiling platform. At the same time, sensitivity analyses were performed to minimise bias due to preclinical disease by excluding those who had been followed for less than two years and who had been diagnosed with CVD at the time of enrolment. However, the present study has several limitations. First, data on healthy lifestyles and heart failure were obtained through self-administered questionnaires at baseline, and the phenotypes or etiologies of heart failure cannot be determined. During the follow-up, some participants may have changed their lifestyles to switch from one exposure group to another. Information about exposures was obtained before the diagnosis of HF, and any misclassification of exposures is likely to be random, associated with HF incidence, and may lead to an underestimation of the magnitude of the association, but the relative magnitude of model performance in outcome assessments improvements (between models with and without metabolic biomarkers) should be largely immune to misclassification. Second, the metabolomic data used in the study were based on NMR nontargeted metabolomic detection of serum samples from the study population, which could not represent the specific metabolomic changes in patients with heart failure. Therefore, we assessed the predictive ability of healthy lifestyle-related metabolites for heart failure risk in addition to traditional risk factors. Furthermore, we did not evaluate variations in the relationship between metabolites and heart failure among diverse demographic groups, including age, sex, and race. Nevertheless, drawing on current mechanistic studies, we can infer that the direction of the association between metabolites and heart failure is likely to remain consistent across various populations. Finally, this study is based on the survey population in the UK Biobank database, and diet and other healthy lifestyles involved in this population may be different from those in other regions, which limits the generalisability of the findings. Whether there are differences in the metabolomics of patients with heart failure in different regions or ethnic groups and whether the results of this study can be extrapolated to other populations remain to be further clarified.

## 5. Conclusions

In summary, we found eight metabolite indicators associated with a healthy lifestyle, three of which, omega-3 fatty acids, DHA, and creatinine, were associated with the risk of heart failure. At the same time, healthy lifestyle-related metabolites can improve the prediction of heart failure risk in addition to traditional risk factors. The results of this study provide a basis for the study of the protective mechanism of a healthy lifestyle on heart failure and emphasise the importance of lifestyle for the prevention of heart failure.

## Figures and Tables

**Table 1:** Baseline characteristics of cohort study participants in the UK Biobank by the incidence of heart failure ^1^.

Baseline Characteristics	Incident Heart Failure			*p* Value
	Total Subjects	Yes	No	
**No. of participants**	121,245	1718	119,527	
**Age (yrs),** mean (SD)	56.5 (8.1)	62.1 (6.1)	56.4 (8.1)	<0.001
**Sex, %**	<0.001			
Male	45.8	66.3	45.6	
Female	54.2	33.7	54.4	
**Race**				0.007
(White, %)	94.3	94.1	94.3	
**Smoking Status, %**	<0.001			
Never	54.5	36.7	54.8	
Past	34.6	45.3	34.4	
Current	10.4	17.3	10.3	
Missing	0.5	0.7	0.5	
**Alcohol drinking, %**	<0.001			
Never	4.3	6.2	4.3	
Past	3.6	8.2	3.5	
Current	91.8	85.2	92.0	
Missing	0.3	0.4	0.2	
**Physical activity**				<0.001
(MET min/week), mean (SD)	2647.4 (2437.6)	2349.7 (2239.8)	2651.7 (2440.1)	
**BMI, %**	<0.001			
Normal (<25 kg/m^2^)	33.1	18.0	33.3	
Overweight (25 to 29.9 kg/m^2^)	42.4	36.9	42.5	
Obesity (≥30 kg/m^2^)	24.2	43.8	23.9	
Missing	0.3	1.3	0.3	
**Townsend deprivation index**	−1.3	−0.5	−1.3	<0.001
**Diabetes, %**				<0.001
Yes	5.2	19.9	5.0	
No	94.4	79.5	94.6	
Missing	0.4	0.6	0.4	
**Hypertension, %**				<0.001
Yes	22.2	73.9	21.5	
No	77.8	26.1	78.5	
**Family history of CVD, %**				<0.001
Yes	56.5	65.1	56.3	
No	36.8	25.6	37.0	
Missing	6.7	9.3	6.7	

^1^ Abbreviations: BMI, body mass index; MET, metabolic equivalent task; CVD, cardiovascular disease. Data are presented as the means ± standard deviations (SDs), numbers and (percentages).

## Data Availability

The data that support the findings of this study are available from the UK Biobank project site, subject to the registration and application process. Further details can be found at https://www.ukbiobank.ac.uk/ (accessed on 11 March 2022).

## Supplementary Materials

The following supporting information can be downloaded at: https://www.mdpi.com/article/10.3390/nu15132934/s1, Figure S1: Pairwise Pearson correlation matrix of 161 metabolites that were included in this study; Figure S2: Random forest algorithm screening in 1:1 matched case-control population; Figure S3: Principal component analysis (PCA) score plot of the 13 healthy lifestyle-related metabolites in a matched case-control; Figure S4: Principal component analysis (PCA) scree plot of the 13 healthy lifestyle-related metabolites in a matched case-control study for heart failure (372 cases and 372 controls); Figure S5: Principal component analysis (PCA) variables plot of the 13 healthy lifestyle-related metabolites; Table S1: Definition and healthy level of each factor in healthy lifestyles; Table S2: Definition and healthy level of each factor in healthy lifestyles; Table S3: Baseline characteristics of 1:1 matched case-control study; Table S4: Linear regression of metabolites and healthy lifestyle in matched case-control study; Table S5: Linear regression of metabolites and healthy lifestyle in cohort study; Table S6: The association of healthy lifestyle-related metabolites with individual components of healthy lifestyle; Table S7: Performance of risk prediction models for incident heart failure; Table S8: Sensitivity analysis.
