# Supplementary material for: The Association of Metabolomic Profiles of a Healthy Lifestyle with Heart Failure Risk in a Prospective Study
Source: Nutrients. 2023 Jun 28;15(13):2934. doi: 10.3390/nu15132934 (PMC10346862; doi:10.3390/nu15132934)
Supplement: Supplementary file 1 [file nutrients-15-02934-s001.zip › nutrients-2450732-supplementary.pdf]

Supplementary Materials

Table of content

Supplementary Figure S1 Pairwise Pearson correlation matrix of 161 metabolites that were included in this study.
Supplementary Figure S2 Random Forest algorithm screening in 1:1 matched case-control population.
Supplementary Figure S3 Principal component analysis (PCA) score plot of the 13 healthy lifestyle-related metabolites in a matched case-control study for heart failure (372 cases and 372 controls).
Supplementary Figure S4 Principal component analysis (PCA) scree plot of the 13 healthy lifestyle-related metabolites in a matched case-control study for heart failure (372 cases and 372 controls).
Supplementary Figure S5 Principal component analysis (PCA) variables plot of the 13 healthy lifestyle-related metabolites in a matched case-control study for heart failure (372 cases and 372 controls).

Supplementary Table S1 Definition and healthy level of each factor in healthy lifestyles.
Supplementary Table S2 Metabolites analyzed in the current study.
Supplementary Table S3 Baseline characteristics of 1:1 matched case-control study.
Supplementary Table S4 Linear regression of metabolites and healthy lifestyle in matched case-control study.
Supplementary Table S5 Linear regression of metabolites and healthy lifestyle in cohort study.
Supplementary Table S6 The association of healthy lifestyle-related metabolites with individual components of healthy lifestyle.
Supplementary Table S7 Performance of risk prediction models for incident heart failure.
Supplementary Table S8 Sensitivity Analysis.

Supplementary Figures

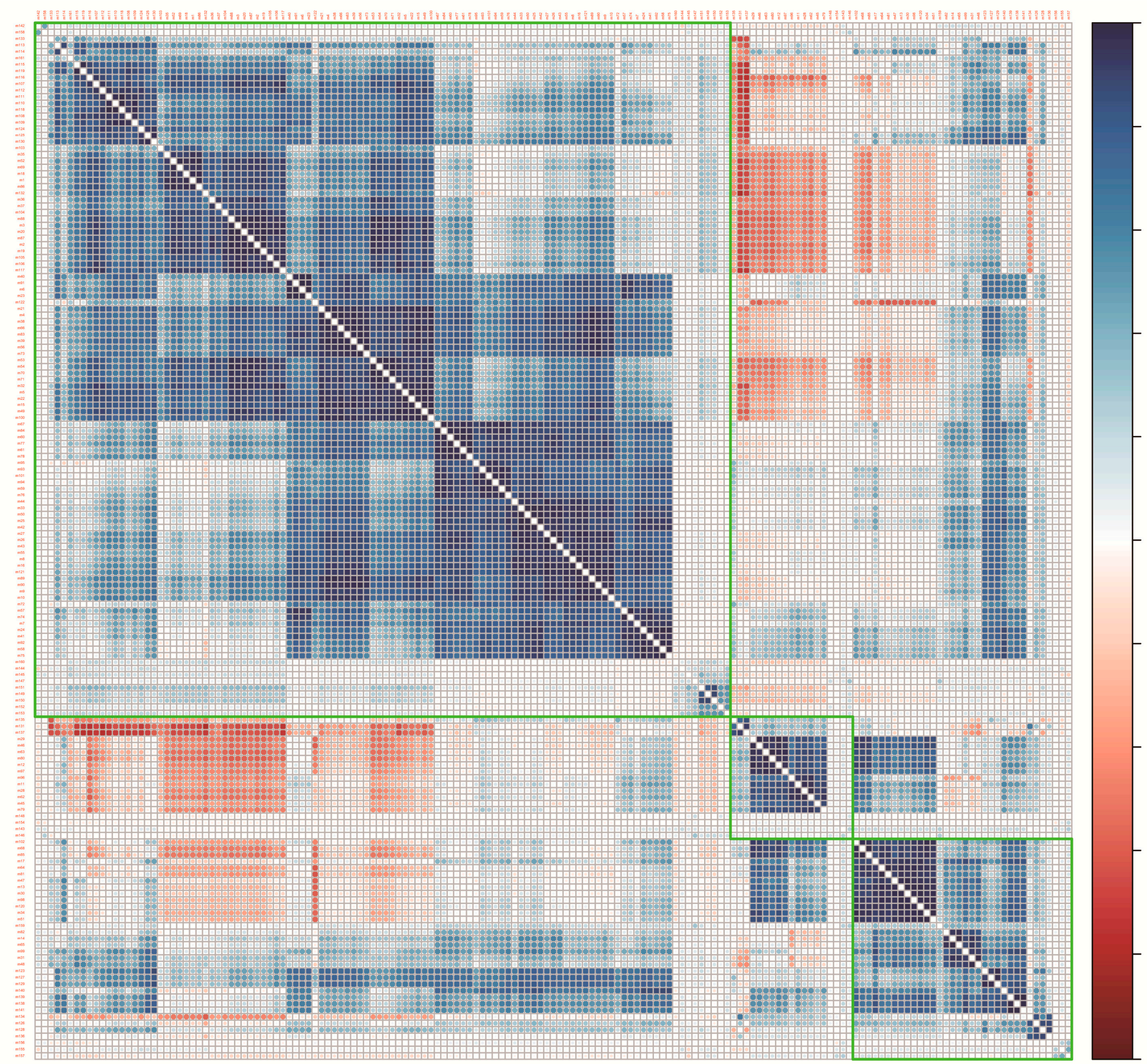

Supplementary Figure S1 Pairwise Pearson correlation matrix of 161 metabolites that were included in this study.

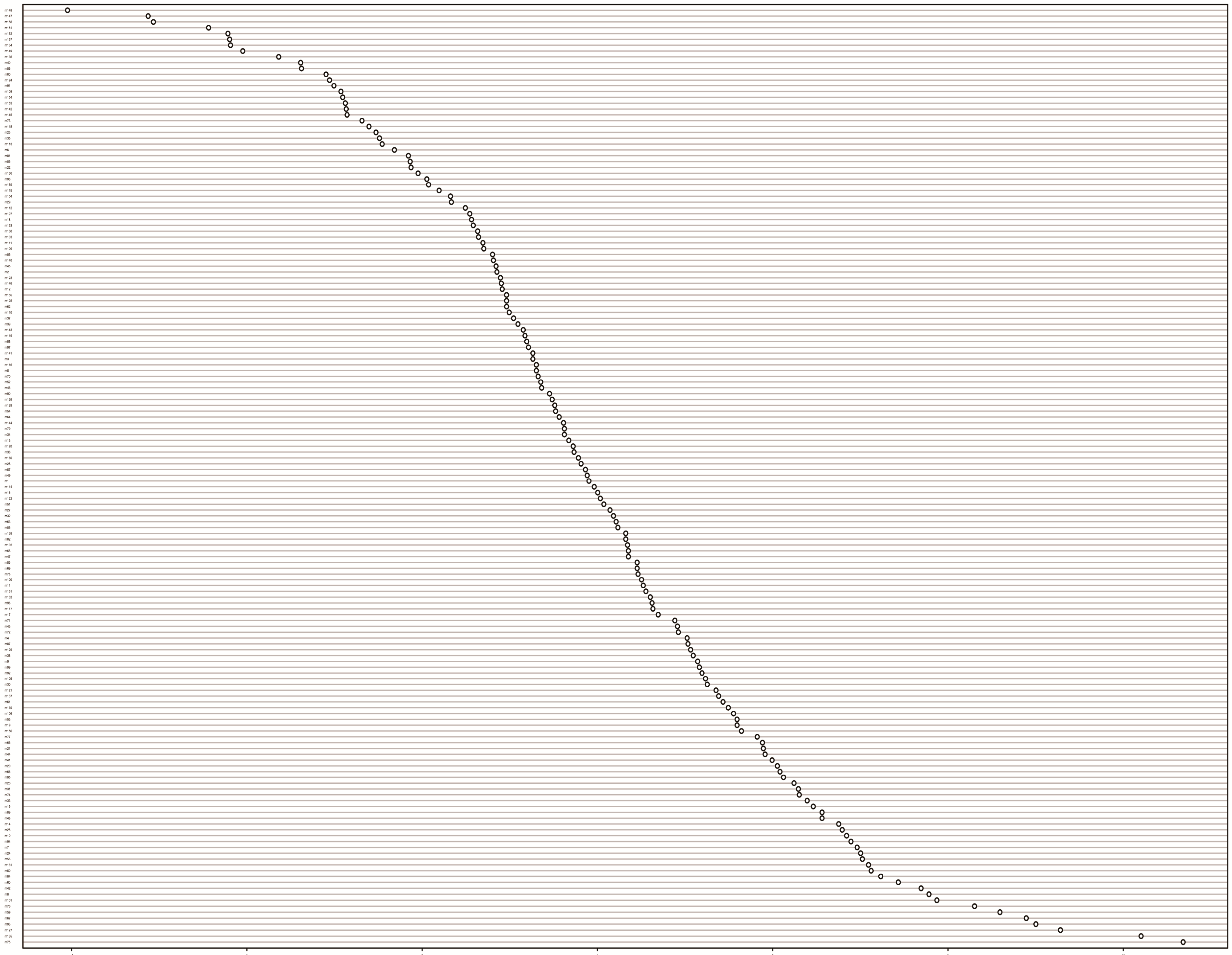

Supplementary Figure S2 Random Forest algorithm screening in 1:1 matched case-control population. The mean decrease accuracy ⩾3 was set as the threshold in the screening. There were 108 metabolites associated with healthy lifestyle were screened by random forest algorithm.

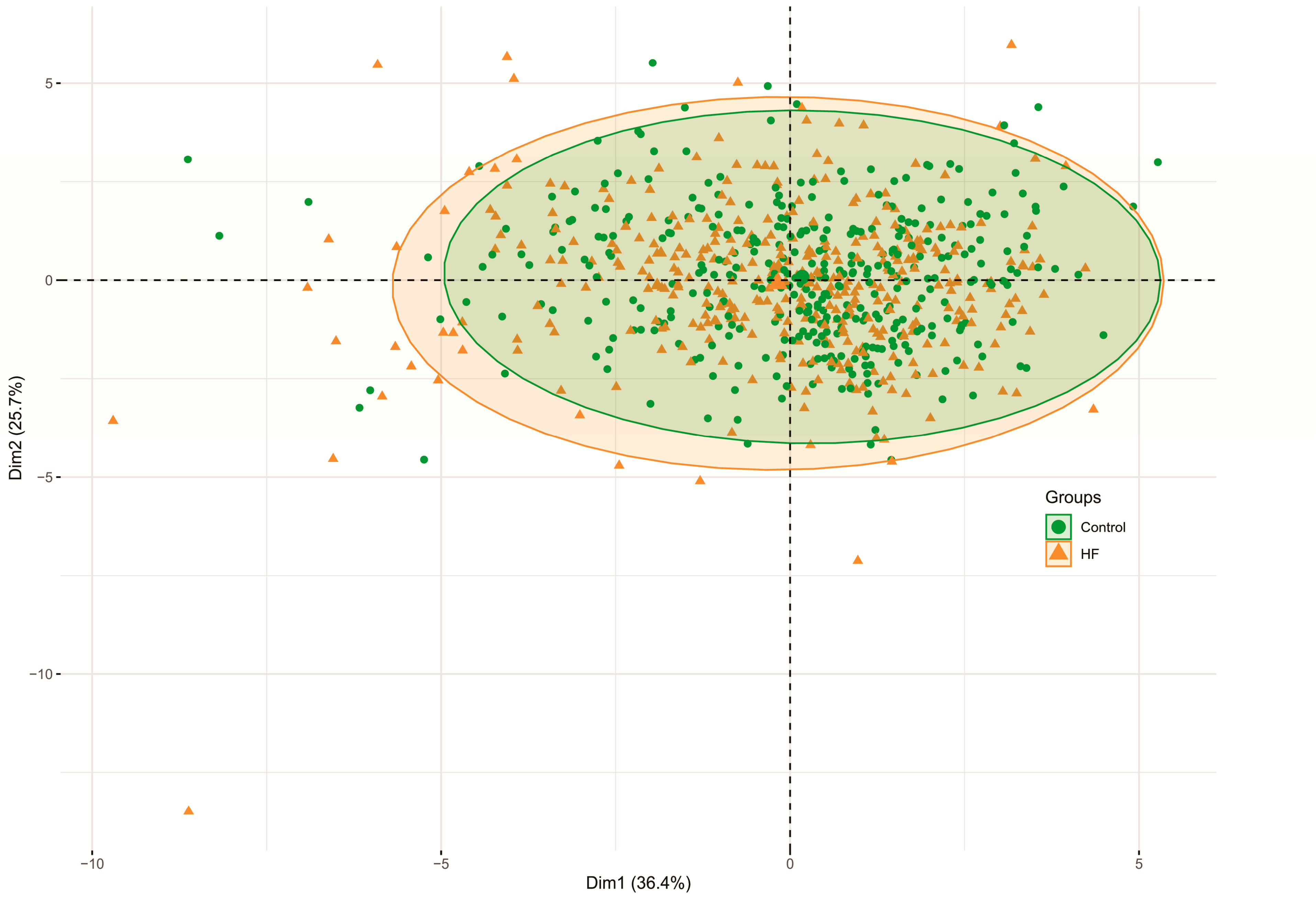

Supplementary Figure S3 Principal component analysis (PCA) score plot of the 13 healthy lifestyle-related metabolites in a matched case-control study for heart failure (372 cases and 372 controls).

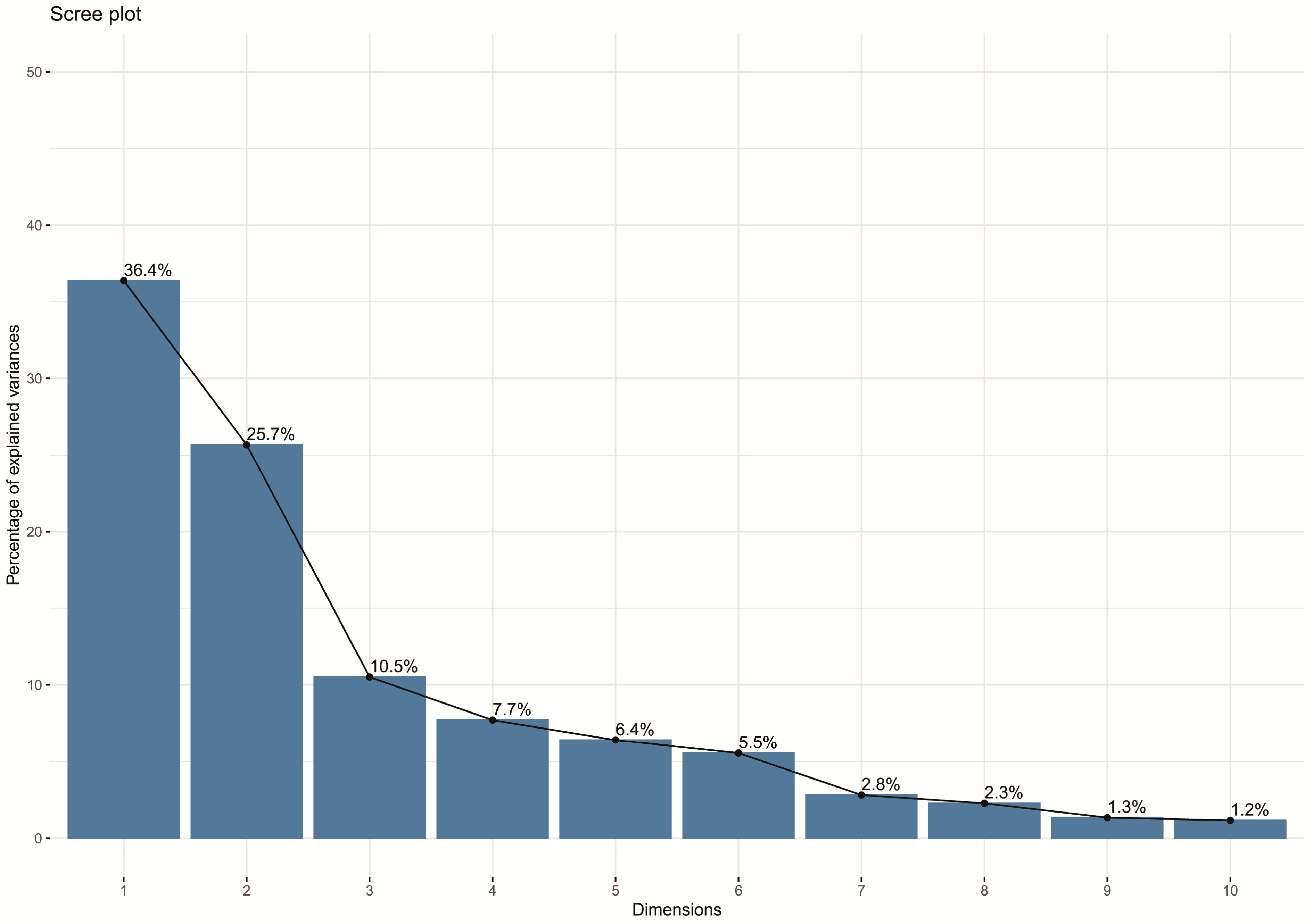

Supplementary Figure S4 Principal component analysis (PCA) scree plot of the 13 healthy lifestyle-related metabolites in a matched case-control study for heart failure (372 cases and 372 controls).

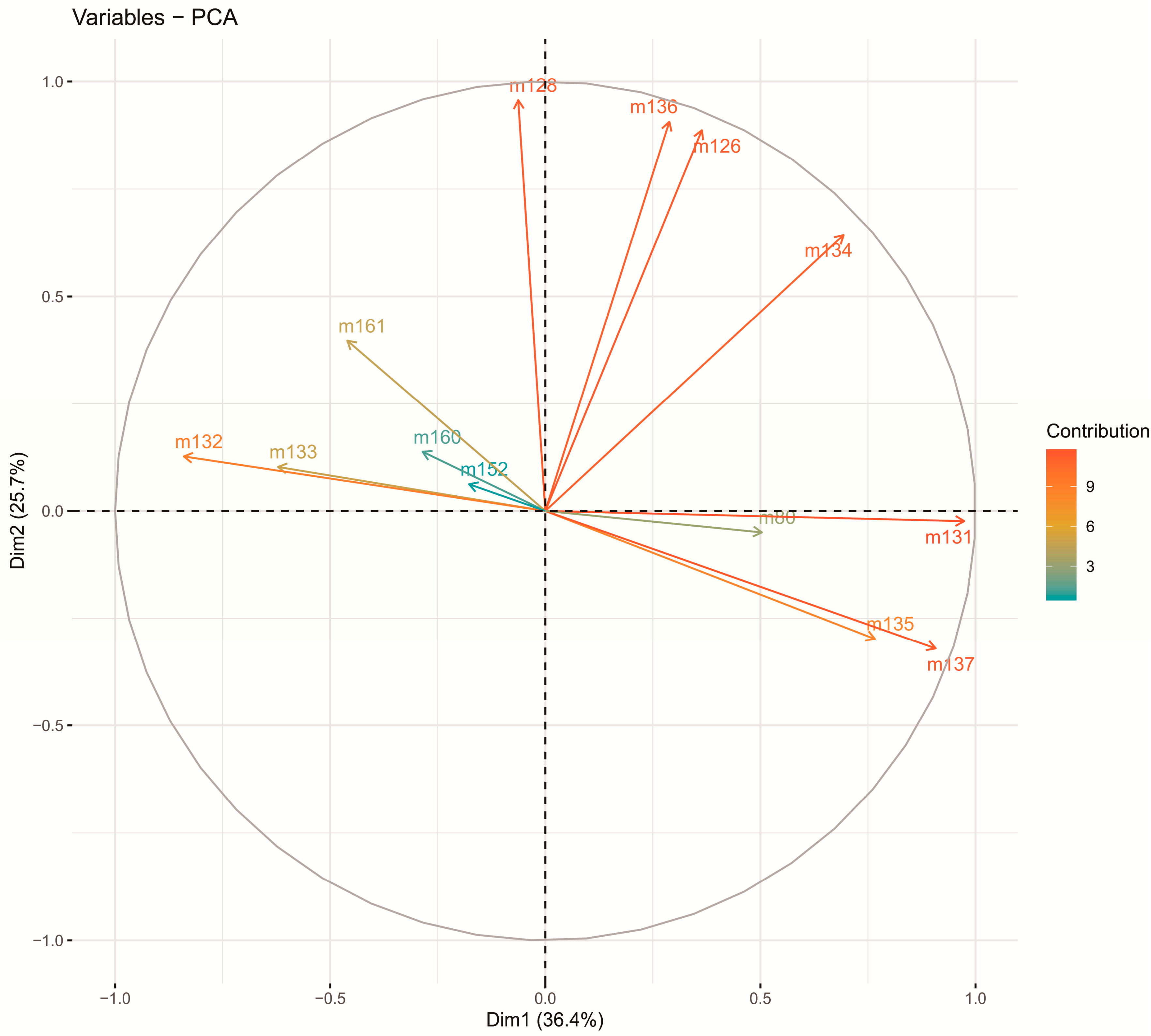

Supplementary Figure S5 Principal component analysis (PCA) variables plot of the 13 healthy lifestyle-related metabolites in a matched case-control study for heart failure (372 cases and 372 controls).

Supplementary Tables

Supplementary Table S1 Definition and healthy level of each factor in healthy lifestyle

**Healthy Lifestyle Factor**	**Healthy Level (Assigned 1 Point)**	**Self-Reported UK Biobank Field Code**
Smoking status	Smoking status was defined as current, previous, never smoker; Never smoking was considered as a healthy level	20116
alcohol consumption	Participants were asked about the frequency of drinking alcohol, i.e., (almost) daily, three or four times a week, once or twice a week, one to three times a month, special occasions only, never, and prefer not to answer. Those who reported to drink alcohol would be asked about how much red wine (glasses), white wine (glasses), beer or cider (pints), spirits or liqueurs (standard measures), fortified wine (glasses), and other alcoholic drinks (glasses) they consumed in an average month or week. We used the information to calculate the average units of alcohol each participant drank daily.<br>A healthy level was defined as daily consumption of one drink or fewer for women and two drinks or fewer for men, according to the dietary guidelines in the US and UK (one drink contains 14 g of ethanol in the US and 8 g in the UK)	20117
physical activity	≥ 150 minutes moderate activity per week OR ≥ 75 minutes vigorous activity per week OR equivalent combination OR moderate physical activity at least 5 days a week and vigorous activity once a week	884, 894, 904, 914
Healthy diet	At least 5 of the following recommendations:<br>1. Fruits: ≥ 3 servings/day<br>2. Vegetables: ≥ 3 servings/day<br>3.Whole grains: ≥ 3 servings/day<br>4. (Shell)Fish: ≥2 times/week<br>5.Diary: ≥ 2 servings/day<br>6.Vegetables oils: ≥ 2 servings/day<br>7.Refined grains: ≤2 servings/day<br>8. Processed meats: ≤ 1 servings/week<br>9. Unprocessed meats: ≤ 2 servings//week<br>10. Sugar-sweetened beverages: Don't drink<br>(Amount per serving: fresh fruit- 1 piece; dried fruit- 5 pieces; vegetables- 3 heaped tablespoons; whole meal/<br>wholegrain bread- 1 slice/day); bran/oat/muesli cereal 1 bowls/week; oily fish/non-oily fish once/week; cheese 1 piece/day; milk type 1 glass/day if consumption of any<br>type of milk; Flora Pro-Active/ Benecol, soft margarine -, olive oil based -, polyunsaturated/sunflower oil based -, other low/reduced fat spread 1 serving/day if in combination with eating at least 2 slices of bread; white, brown, other bread slices 1 slice/day; biscuit, other cereals 1 bowl/day; processed meat 1 piece/day; 0 pieces/day if indicated having never eaten meat; poultry/beef/lamb or mutton/pork once/week.	1309, 1319, 1289, 1299, 1438,1448,1458,1468, 1329,1339,1408,1418, 1428,2654,1349,3680, 1359,1369,1379,1389,6144

Supplementary Table S2 Metabolites analyzed in the current study

ID	Metabolites	Superclass
m1	Concentration of Chylomicrons and Extremely Large VLDL Particles	Lipoprotein particle concentrations
m2	Concentration of Very Large VLDL Particles	Lipoprotein particle concentrations
m3	Concentration of Large VLDL Particles	Lipoprotein particle concentrations
m4	Concentration of Medium VLDL Particles	Lipoprotein particle concentrations
m5	Concentration of Small VLDL Particles	Lipoprotein particle concentrations
m6	Concentration of Very Small VLDL Particles	Lipoprotein particle concentrations
m7	Concentration of IDL Particles	Lipoprotein particle concentrations
m8	Concentration of Large LDL Particles	Lipoprotein particle concentrations
m9	Concentration of Medium LDL Particles	Lipoprotein particle concentrations
m10	Concentration of Small LDL Particles	Lipoprotein particle concentrations
m11	Concentration of Very Large HDL Particles	Lipoprotein particle concentrations
m12	Concentration of Large HDL Particles	Lipoprotein particle concentrations
m13	Concentration of Medium HDL Particles	Lipoprotein particle concentrations
m14	Concentration of Small HDL Particles	Lipoprotein particle concentrations
m15	Concentration of VLDL Particles	Lipoprotein particle concentrations
m16	Concentration of LDL Particles	Lipoprotein particle concentrations
m17	Concentration of HDL Particles	Lipoprotein particle concentrations
m18	Total Lipids in Chylomicrons and Extremely Large VLDL	Total lipid concentrations
m19	Total Lipids in Very Large VLDL	Total lipid concentrations
m20	Total Lipids in Large VLDL	Total lipid concentrations
m21	Total Lipids in Medium VLDL	Total lipid concentrations
m22	Total Lipids in Small VLDL	Total lipid concentrations
m23	Total Lipids in Very Small VLDL	Total lipid concentrations
m24	Total Lipids in IDL	Total lipid concentrations
m25	Total Lipids in Large LDL	Total lipid concentrations
m26	Total Lipids in Medium LDL	Total lipid concentrations
m27	Total Lipids in Small LDL	Total lipid concentrations
m28	Total Lipids in Very Large HDL	Total lipid concentrations
m29	Total Lipids in Large HDL	Total lipid concentrations
m30	Total Lipids in Medium HDL	Total lipid concentrations
m31	Total Lipids in Small HDL	Total lipid concentrations
m32	Total Lipids in VLDL	Total lipid concentrations
m33	Total Lipids in LDL	Total lipid concentrations
m34	Total Lipids in HDL	Total lipid concentrations
m35	Phospholipids in Chylomicrons and Extremely Large VLDL	Phospholipid concentrations
m36	Phospholipids in Very Large VLDL	Phospholipid concentrations
m37	Phospholipids in Large VLDL	Phospholipid concentrations
m38	Phospholipids in Medium VLDL	Phospholipid concentrations
m39	Phospholipids in Small VLDL	Phospholipid concentrations
m40	Phospholipids in Very Small VLDL	Phospholipid concentrations
m41	Phospholipids in IDL	Phospholipid concentrations
m42	Phospholipids in Large LDL	Phospholipid concentrations
m43	Phospholipids in Medium LDL	Phospholipid concentrations
m44	Phospholipids in Small LDL	Phospholipid concentrations
m45	Phospholipids in Very Large HDL	Phospholipid concentrations
m46	Phospholipids in Large HDL	Phospholipid concentrations
m47	Phospholipids in Medium HDL	Phospholipid concentrations
m48	Phospholipids in Small HDL	Phospholipid concentrations
m49	Phospholipids in VLDL	Phospholipid concentrations
m50	Phospholipids in LDL	Phospholipid concentrations

m51	Phospholipids in HDL	Phospholipid concentrations
m52	Cholesterol in Chylomicrons and Extremely Large VLDL	Cholesterol concentrations
m53	Cholesterol in Very Large VLDL	Cholesterol concentrations
m54	Cholesterol in Large VLDL	Cholesterol concentrations
m55	Cholesterol in Medium VLDL	Cholesterol concentrations
m56	Cholesterol in Small VLDL	Cholesterol concentrations
m57	Cholesterol in Very Small VLDL	Cholesterol concentrations
m58	Cholesterol in IDL	Cholesterol concentrations
m59	Cholesterol in Large LDL	Cholesterol concentrations
m60	Cholesterol in Medium LDL	Cholesterol concentrations
m61	Cholesterol in Small LDL	Cholesterol concentrations
m62	Cholesterol in Very Large HDL	Cholesterol concentrations
m63	Cholesterol in Large HDL	Cholesterol concentrations
m64	Cholesterol in Medium HDL	Cholesterol concentrations
m65	Cholesterol in Small HDL	Cholesterol concentrations
m66	VLDL Cholesterol	Cholesterol concentrations
m67	LDL Cholesterol	Cholesterol concentrations
m68	HDL Cholesterol	Cholesterol concentrations
m69	Cholesteryl Esters in Chylomicrons and Extremely Large VLDL	Esterified cholesterol concentrations
m70	Cholesteryl Esters in Very Large VLDL	Esterified cholesterol concentrations
m71	Cholesteryl Esters in Large VLDL	Esterified cholesterol concentrations
m72	Cholesteryl Esters in Medium VLDL	Esterified cholesterol concentrations
m73	Cholesteryl Esters in Small VLDL	Esterified cholesterol concentrations
m74	Cholesteryl Esters in Very Small VLDL	Esterified cholesterol concentrations
m75	Cholesteryl Esters in IDL	Esterified cholesterol concentrations
m76	Cholesteryl Esters in Large LDL	Esterified cholesterol concentrations
m77	Cholesteryl Esters in Medium LDL	Esterified cholesterol concentrations
m78	Cholesteryl Esters in Small LDL	Esterified cholesterol concentrations
m79	Cholesteryl Esters in Very Large HDL	Esterified cholesterol concentrations
m80	Cholesteryl Esters in Large HDL	Esterified cholesterol concentrations
m81	Cholesteryl Esters in Medium HDL	Esterified cholesterol concentrations
m82	Cholesteryl Esters in Small HDL	Esterified cholesterol concentrations
m83	Cholesteryl Esters in VLDL	Esterified cholesterol concentrations
m84	Cholesteryl Esters in LDL	Esterified cholesterol concentrations
m85	Cholesteryl Esters in HDL	Esterified cholesterol concentrations
m86	Free Cholesterol in Chylomicrons and Extremely Large VLDL	Free cholesterol concentrations
m87	Free Cholesterol in Very Large VLDL	Free cholesterol concentrations
m88	Free Cholesterol in Large VLDL	Free cholesterol concentrations
m89	Free Cholesterol in Medium VLDL	Free cholesterol concentrations
m90	Free Cholesterol in Small VLDL	Free cholesterol concentrations
m91	Free Cholesterol in Very Small VLDL	Free cholesterol concentrations
m92	Free Cholesterol in IDL	Free cholesterol concentrations
m93	Free Cholesterol in Large LDL	Free cholesterol concentrations
m94	Free Cholesterol in Medium LDL	Free cholesterol concentrations
m95	Free Cholesterol in Small LDL	Free cholesterol concentrations
m96	Free Cholesterol in Very Large HDL	Free cholesterol concentrations
m97	Free Cholesterol in Large HDL	Free cholesterol concentrations
m98	Free Cholesterol in Medium HDL	Free cholesterol concentrations
m99	Free Cholesterol in Small HDL	Free cholesterol concentrations
m100	Free Cholesterol in VLDL	Free cholesterol concentrations
m101	Free Cholesterol in LDL	Free cholesterol concentrations
m102	Free Cholesterol in HDL	Free cholesterol concentrations
m103	Triglycerides in Chylomicrons and Extremely Large VLDL	Triglyceride concentrations

m104	Triglycerides in Very Large VLDL	Triglyceride concentrations
m105	Triglycerides in Large VLDL	Triglyceride concentrations
m106	Triglycerides in Medium VLDL	Triglyceride concentrations
m107	Triglycerides in Small VLDL	Triglyceride concentrations
m108	Triglycerides in Very Small VLDL	Triglyceride concentrations
m109	Triglycerides in IDL	Triglyceride concentrations
m110	Triglycerides in Large LDL	Triglyceride concentrations
m111	Triglycerides in Medium LDL	Triglyceride concentrations
m112	Triglycerides in Small LDL	Triglyceride concentrations
m113	Triglycerides in Very Large HDL	Triglyceride concentrations
m114	Triglycerides in Large HDL	Triglyceride concentrations
m115	Triglycerides in Medium HDL	Triglyceride concentrations
m116	Triglycerides in Small HDL	Triglyceride concentrations
m117	Triglycerides in VLDL	Triglyceride concentrations
m118	Triglycerides in LDL	Triglyceride concentrations
m119	Triglycerides in HDL	Triglyceride concentrations
m120	Apolipoprotein A1	Apolipoproteins
m121	Apolipoprotein B	Apolipoproteins
m122	Apolipoprotein B to Apolipoprotein A1 ratio	Apolipoproteins
m123	Polyunsaturated Fatty Acids	Fatty acids
m124	Monounsaturated Fatty Acids	Fatty acids
m125	Saturated Fatty Acids	Fatty acids
m126	Docosahexaenoic Acid	Fatty acids
m127	Linoleic Acid	Fatty acids
m128	Omega-3 Fatty Acids	Fatty acids
m129	Omega-6 Fatty Acids	Fatty acids
m130	Total Fatty Acids	Fatty acids
m131	Polyunsaturated Fatty Acids to Total Fatty Acids percentage	Fatty acids
m132	Monounsaturated Fatty Acids to Total Fatty Acids percentage	Fatty acids
m133	Saturated Fatty Acids to Total Fatty Acids percentage	Fatty acids
m134	Docosahexaenoic Acid to Total Fatty Acids percentage	Fatty acids
m135	Linoleic Acid to Total Fatty Acids percentage	Fatty acids
m136	Omega-3 Fatty Acids to Total Fatty Acids percentage	Fatty acids
m137	Omega-6 Fatty Acids to Total Fatty Acids percentage	Fatty acids
m138	Total Cholines	Other lipids
m139	Phosphatidylcholines	Other lipids
m140	Sphingomyelins	Other lipids
m141	Phosphoglycerides	Other lipids
m142	Lactate	Glycolysis-related metabolites
m143	Citrate	Glycolysis-related metabolites
m144	Glucose	Glycolysis-related metabolites
m145	Alanine	Amino acids
m146	Glutamine	Amino acids
m147	Histidine	Amino acids
m148	Glycine	Amino acids
m149	Isoleucine	Amino acids
m150	Leucine	Amino acids
m151	Valine	Amino acids
m152	Phenylalanine	Amino acids
m153	Tyrosine	Amino acids
m154	Acetate	Ketone bodies
m155	Acetoacetate	Ketone bodies
m156	3-Hydroxybutyrate	Ketone bodies

m157	Acetone	Ketone bodies
m158	Pyruvate	Ketone bodies
m159	Albumin	Fluid balance
m160	Creatinine	Fluid balance
m161	Glycoprotein Acetyls	Inflammation

Supplementary Table S3 Baseline characteristics of 1:1 matched case-control study

**Baseline Characteristics***	**Control N=372**	**Case N=372**	**Bias, %**	***p* Value**
**Age(yrs),** mean (SD)	60.2 (7.2)	61.0 (6.6)	2.1	0.749
**Sex, %**			−6.5	0.309
Male	80.1	78.8		
Female	19.9	21.2		
**Race**			2.1	0.782
(White, %)	91.9	94.1		
**Smoking Status, %**			0.2	0.977
Never	46.3	32.7		
Past	44.9	55.0		
Current	8.3	11.8		
Missing	0.5	0.5		
**Alcohol drinking, %**			−0.1	0.598
Never	5.9	4.5		
Past	5.7	7.8		
Current	88.4	87.7		
**Physical activity** (MET Min/ week), mean (SD)	2098.3 (2287.2)	2267.6 (2502.6)	−1.3	0.871
**BMI, %**			3.3	0.648
Normal (<25 kg/m^2)	20.5	19.8		
Overweight (25 to 29.9 kg/m^2)	43.0	35.7		
Obesity (≥30 kg/m^2)	36.0	43.4		
Missing	0.5	1.1		
**Townsend deprivation index**	−0.62 (3.4)	−0.58 (3.4)	0.2	0.979
**Diabetes, %**			−8.6	0.325
Yes	9.4	21.2		
No	89.8	78.6		
Missing	0.8	0.2		
**Hypertension, %**			2.5	0.742
Yes	74.2	73.5		
No	25.8	26.5		
**Family history of CVD, %**			0.0	0.999
Yes	61.9	73.5		
No	33.3	22.0		
Missing	4.8	4.5		

* Mean (SD) is presented for continuous variables and percentage for categorical variables.

Supplementary Table S4 Linear regression of metabolites and healthy lifestyle in matched case-control study[1]

Metabolites	Super Class	Coef..	95% CI	*p*
Concentration of Chylomicrons and Extremely Large VLDL Particles	Lipoprotein particle concentrations	−0.019	(−0.063, 0.025)	0.391
Concentration of Very Large VLDL Particles	Lipoprotein particle concentrations	−0.017	(−0.047, 0.012)	0.245
Concentration of Large VLDL Particles	Lipoprotein particle concentrations	−0.01	(−0.033, 0.012)	0.372
Concentration of Medium VLDL Particles	Lipoprotein particle concentrations	−0.001	(−0.017, 0.016)	0.937
Concentration of Small VLDL Particles	Lipoprotein particle concentrations	−0.003	(−0.017, 0.01)	0.625
Concentration of Very Small VLDL Particles	Lipoprotein particle concentrations	−0.003	(−0.014, 0.007)	0.532
Concentration of IDL Particles	Lipoprotein particle concentrations	−0.001	(−0.012, 0.01)	0.805
Concentration of Large LDL Particles	Lipoprotein particle concentrations	0	(−0.011, 0.011)	0.996
Concentration of Medium LDL Particles	Esterified cholesterol concentrations	−0.001	(−0.013, 0.01)	0.862
Concentration of Small LDL Particles	Lipoprotein particle concentrations	−0.002	(−0.011, 0.008)	0.742
Concentration of Very Large HDL Particles	Lipoprotein particle concentrations	0.008	(−0.005, 0.022)	0.231
Concentration of Large HDL Particles	Lipoprotein particle concentrations	0.022	(−0.001, 0.045)	0.062
Concentration of Medium HDL Particles	Lipoprotein particle concentrations	0.004	(−0.006, 0.014)	0.424
Concentration of Small HDL Particles	Lipoprotein particle concentrations	0.001	(−0.005, 0.007)	0.801
Concentration of VLDL Particles	Lipoprotein particle concentrations	−0.004	(−0.017, 0.009)	0.571
Concentration of LDL Particles	Lipoprotein particle concentrations	−0.001	(−0.011, 0.01)	0.923
Concentration of HDL Particles	Lipoprotein particle concentrations	0.003	(−0.004, 0.009)	0.45
Total Lipids in Chylomicrons and Extremely Large VLDL	Lipoprotein particle concentrations	−0.019	(−0.06, 0.022)	0.361
Total Lipids in Very Large VLDL	Total lipid concentrations	−0.017	(−0.046, 0.012)	0.262
Total Lipids in Large VLDL	Total lipid concentrations	−0.009	(−0.032, 0.014)	0.432
Total Lipids in Medium VLDL	Total lipid concentrations	−0.002	(−0.019, 0.015)	0.829
Total Lipids in Small VLDL	Total lipid concentrations	−0.003	(−0.016, 0.011)	0.682
Total Lipids in Very Small VLDL	Total lipid concentrations	−0.003	(−0.014, 0.007)	0.548
Total Lipids in IDL	Total lipid concentrations	0.001	(−0.01, 0.011)	0.918
Total Lipids in Large LDL	Total lipid concentrations	−0.001	(−0.012, 0.011)	0.903
Total Lipids in Medium LDL	Total lipid concentrations	−0.002	(−0.015, 0.01)	0.716
Total Lipids in Small LDL	Total lipid concentrations	−0.002	(−0.013, 0.008)	0.676
Total Lipids in Very Large HDL	Total lipid concentrations	0.011	(−0.007, 0.028)	0.225
Total Lipids in Large HDL	Total lipid concentrations	0.016	(−0.002, 0.034)	0.076
Total Lipids in Medium HDL	Total lipid concentrations	0.003	(−0.006, 0.012)	0.528
Total Lipids in Small HDL	Total lipid concentrations	0	(−0.006, 0.006)	0.893
Total Lipids in VLDL	Total lipid concentrations	−0.007	(−0.024, 0.01)	0.43
Total Lipids in LDL	Total lipid concentrations	−0.001	(−0.013, 0.01)	0.824
Total Lipids in HDL	Total lipid concentrations	0.004	(−0.004, 0.011)	0.373
Phospholipids in Chylomicrons and Extremely Large VLDL	Total lipid concentrations	−0.013	(−0.065, 0.04)	0.637
Phospholipids in Very Large VLDL	Phospholipid concentrations	−0.022	(−0.061, 0.017)	0.261
Phospholipids in Large VLDL	Phospholipid concentrations	−0.027	(−0.067, 0.014)	0.192
Phospholipids in Medium VLDL	Phospholipid concentrations	−0.001	(−0.019, 0.018)	0.933
Phospholipids in Small VLDL	Phospholipid concentrations	−0.001	(−0.015, 0.013)	0.915
Phospholipids in Very Small VLDL	Phospholipid concentrations	−0.004	(−0.015, 0.006)	0.43
Phospholipids in IDL	Phospholipid concentrations	−0.001	(−0.011, 0.009)	0.892
Phospholipids in Large LDL	Phospholipid concentrations	0	(−0.011, 0.011)	0.985
Phospholipids in Medium LDL	Phospholipid concentrations	−0.002	(−0.014, 0.01)	0.751
Phospholipids in Small LDL	Phospholipid concentrations	−0.002	(−0.011, 0.008)	0.688
Phospholipids in Very Large HDL	Phospholipid concentrations	0.009	(−0.02, 0.038)	0.549
Phospholipids in Large HDL	Phospholipid concentrations	0.013	(−0.004, 0.03)	0.123
Phospholipids in Medium HDL	Phospholipid concentrations	0.002	(−0.007, 0.01)	0.683
Phospholipids in Small HDL	Phospholipid concentrations	−0.001	(−0.007, 0.005)	0.788
Phospholipids in VLDL	Phospholipid concentrations	−0.006	(−0.023, 0.011)	0.484
Phospholipids in LDL	Phospholipid concentrations	−0.001	(−0.012, 0.01)	0.874

Phospholipids in HDL	Phospholipid concentrations	0.002	(−0.005, 0.01)	0.526
Cholesterol in Chylomicrons and Extremely Large VLDL	Phospholipid concentrations	−0.013	(−0.046, 0.02)	0.429
Cholesterol in Very Large VLDL	Cholesterol concentrations	−0.011	(−0.037, 0.015)	0.403
Cholesterol in Large VLDL	Cholesterol concentrations	−0.007	(−0.03, 0.015)	0.522
Cholesterol in Medium VLDL	Cholesterol concentrations	0.007	(−0.014, 0.028)	0.512
Cholesterol in Small VLDL	Cholesterol concentrations	−0.002	(−0.016, 0.013)	0.821
Cholesterol in Very Small VLDL	Cholesterol concentrations	−0.001	(−0.013, 0.011)	0.879
Cholesterol in IDL	Cholesterol concentrations	0.002	(−0.01, 0.014)	0.72
Cholesterol in Large LDL	Cholesterol concentrations	0.001	(−0.011, 0.013)	0.811
Cholesterol in Medium LDL	Cholesterol concentrations	−0.021	(−0.063, 0.021)	0.335
Cholesterol in Small LDL	Cholesterol concentrations	−0.02	(−0.059, 0.02)	0.328
Cholesterol in Very Large HDL	Cholesterol concentrations	0.011	(−0.004, 0.026)	0.16
Cholesterol in Large HDL	Cholesterol concentrations	0.025	(−0.001, 0.05)	0.059
Cholesterol in Medium HDL	Cholesterol concentrations	0.005	(−0.005, 0.015)	0.293
Cholesterol in Small HDL	Cholesterol concentrations	0.001	(−0.005, 0.007)	0.66
VLDL Cholesterol	Cholesterol concentrations	−0.003	(−0.018, 0.013)	0.708
LDL Cholesterol	Cholesterol concentrations	−0.02	(−0.063, 0.023)	0.356
HDL Cholesterol	Cholesterol concentrations	0.006	(−0.002, 0.015)	0.159
Cholesteryl Esters in Chylomicrons and Extremely Large VLDL	Cholesterol concentrations	−0.017	(−0.051, 0.018)	0.35
Cholesteryl Esters in Very Large VLDL	Esterified cholesterol concentrations	−0.01	(−0.035, 0.016)	0.467
Cholesteryl Esters in Large VLDL	Esterified cholesterol concentrations	−0.004	(−0.026, 0.018)	0.714
Cholesteryl Esters in Medium VLDL	Esterified cholesterol concentrations	0.026	(−0.01, 0.061)	0.156
Cholesteryl Esters in Small VLDL	Esterified cholesterol concentrations	−0.003	(−0.018, 0.012)	0.684
Cholesteryl Esters in Very Small VLDL	Esterified cholesterol concentrations	0	(−0.012, 0.012)	0.986
Cholesteryl Esters in IDL	Esterified cholesterol concentrations	0.002	(−0.01, 0.014)	0.696
Cholesteryl Esters in Large LDL	Esterified cholesterol concentrations	0.001	(−0.011, 0.013)	0.842
Cholesteryl Esters in Medium LDL	Esterified cholesterol concentrations	−0.001	(−0.015, 0.013)	0.867
Cholesteryl Esters in Small LDL	Esterified cholesterol concentrations	−0.021	(−0.061, 0.02)	0.313
Cholesteryl Esters in Very Large HDL	Esterified cholesterol concentrations	0.015	(−0.003, 0.033)	0.109
Cholesteryl Esters in Large HDL	Esterified cholesterol concentrations	0.026	(0.001, 0.052)	0.043 *
Cholesteryl Esters in Medium HDL	Esterified cholesterol concentrations	0.005	(−0.004, 0.015)	0.276
Cholesteryl Esters in Small HDL	Esterified cholesterol concentrations	0.002	(−0.004, 0.008)	0.54
Cholesteryl Esters in VLDL	Esterified cholesterol concentrations	−0.002	(−0.017, 0.013)	0.794
Cholesteryl Esters in LDL	Esterified cholesterol concentrations	−0.022	(−0.067, 0.023)	0.343
Cholesteryl Esters in HDL	Esterified cholesterol concentrations	0.007	(−0.002, 0.016)	0.132
Free Cholesterol in Chylomicrons and Extremely Large VLDL	Free cholesterol concentrations	−0.01	(−0.044, 0.023)	0.542
Free Cholesterol in Very Large VLDL	Free cholesterol concentrations	−0.015	(−0.043, 0.013)	0.285
Free Cholesterol in Large VLDL	Free cholesterol concentrations	−0.01	(−0.034, 0.013)	0.391
Free Cholesterol in Medium VLDL	Free cholesterol concentrations	0.003	(−0.016, 0.022)	0.791
Free Cholesterol in Small VLDL	Free cholesterol concentrations	0.001	(−0.014, 0.016)	0.927
Free Cholesterol in Very Small VLDL	Free cholesterol concentrations	−0.003	(−0.013, 0.008)	0.651
Free Cholesterol in IDL	Free cholesterol concentrations	0.001	(−0.01, 0.013)	0.81
Free Cholesterol in Large LDL	Free cholesterol concentrations	0.002	(−0.011, 0.015)	0.76
Free Cholesterol in Medium LDL	Free cholesterol concentrations	−0.018	(−0.058, 0.023)	0.391
Free Cholesterol in Small LDL	Free cholesterol concentrations	−0.017	(−0.056, 0.021)	0.378
Free Cholesterol in Very Large HDL	Free cholesterol concentrations	0.003	(−0.008, 0.014)	0.556
Free Cholesterol in Large HDL	Free cholesterol concentrations	0.02	(−0.009, 0.05)	0.182
Free Cholesterol in Medium HDL	Free cholesterol concentrations	0.005	(−0.006, 0.017)	0.371
Free Cholesterol in Small HDL	Free cholesterol concentrations	0	(−0.006, 0.006)	0.979
Free Cholesterol in VLDL	Free cholesterol concentrations	−0.004	(−0.02, 0.012)	0.619
Free Cholesterol in LDL	Free cholesterol concentrations	−0.018	(−0.059, 0.023)	0.388
Free Cholesterol in HDL	Free cholesterol concentrations	0.005	(−0.004, 0.013)	0.317
Triglycerides in Chylomicrons and Extremely Large VLDL	Triglyceride concentrations	−0.026	(−0.091, 0.04)	0.447

Triglycerides in Very Large VLDL	Triglyceride concentrations	−0.02	(−0.052, 0.011)	0.208
Triglycerides in Large VLDL	Triglyceride concentrations	−0.009	(−0.032, 0.014)	0.456
Triglycerides in Medium VLDL	Triglyceride concentrations	−0.005	(−0.022, 0.013)	0.615
Triglycerides in Small VLDL	Triglyceride concentrations	−0.005	(−0.02, 0.01)	0.543
Triglycerides in Very Small VLDL	Triglyceride concentrations	−0.007	(−0.019, 0.005)	0.253
Triglycerides in IDL	Triglyceride concentrations	−0.007	(−0.018, 0.003)	0.183
Triglycerides in Large LDL	Triglyceride concentrations	−0.008	(−0.019, 0.003)	0.147
Triglycerides in Medium LDL	Triglyceride concentrations	−0.009	(−0.022, 0.004)	0.184
Triglycerides in Small LDL	Triglyceride concentrations	−0.009	(−0.023, 0.006)	0.244
Triglycerides in Very Large HDL	Triglyceride concentrations	−0.005	(−0.021, 0.011)	0.541
Triglycerides in Large HDL	Triglyceride concentrations	−0.002	(−0.018, 0.014)	0.799
Triglycerides in Medium HDL	Triglyceride concentrations	−0.007	(−0.021, 0.007)	0.304
Triglycerides in Small HDL	Triglyceride concentrations	−0.009	(−0.021, 0.004)	0.162
Triglycerides in VLDL	Triglyceride concentrations	−0.01	(−0.031, 0.011)	0.349
Triglycerides in LDL	Triglyceride concentrations	−0.008	(−0.02, 0.003)	0.161
Triglycerides in HDL	Triglyceride concentrations	−0.007	(−0.02, 0.006)	0.289
Apolipoprotein A1	Apolipoproteins	0.002	(−0.004, 0.009)	0.472
Apolipoprotein B	Apolipoproteins	−0.001	(−0.011, 0.01)	0.856
Apolipoprotein B to Apolipoprotein A1 ratio	Apolipoproteins	−0.003	(−0.015, 0.008)	0.57
Polyunsaturated Fatty Acids	Fatty acids	0.005	(−0.002, 0.012)	0.14
Monounsaturated Fatty Acids	Fatty acids	−0.008	(−0.021, 0.005)	0.215
Saturated Fatty Acids	Fatty acids	−0.006	(−0.017, 0.004)	0.256
Docosahexaenoic Acid	Fatty acids	0.021	(0.006, 0.036)	0.005 *
Linoleic Acid	Fatty acids	0.004	(−0.005, 0.013)	0.35
Omega-3 Fatty Acids	Fatty acids	0.02	(0.003, 0.037)	0.021 *
Omega-6 Fatty Acids	Fatty acids	0.003	(−0.004, 0.01)	0.359
Total Fatty Acids	Fatty acids	−0.002	(−0.011, 0.007)	0.677
Polyunsaturated Fatty Acids to Total Fatty Acids percentage	Fatty acids	0.007	(0.003, 0.012)	0.001 *
Monounsaturated Fatty Acids to Total Fatty Acids percentage	Fatty acids	−0.006	(−0.011, −0.001)	0.01 *
Saturated Fatty Acids to Total Fatty Acids percentage	Fatty acids	−0.004	(−0.007, −0.002)	0.002 *
Docosahexaenoic Acid to Total Fatty Acids percentage	Fatty acids	0.023	(0.008, 0.038)	0.002 *
Linoleic Acid to Total Fatty Acids percentage	Fatty acids	0.006	(0.001, 0.012)	0.024 *
Omega-3 Fatty Acids to Total Fatty Acids percentage	Fatty acids	0.022	(0.009, 0.035)	0.001 *
Omega-6 Fatty Acids to Total Fatty Acids percentage	Fatty acids	0.005	(0, 0.01)	0.035 *
Total Cholines	Other lipids	0.001	(−0.006, 0.008)	0.721
Phosphatidylcholines	Other lipids	0.002	(−0.006, 0.009)	0.704
Sphingomyelins	Other lipids	−0.001	(−0.007, 0.006)	0.858
Phosphoglycerides	Other lipids	0.001	(−0.007, 0.009)	0.781
Lactate	Glycolysis-related metabolites	−0.005	(−0.017, 0.008)	0.459
Citrate	Glycolysis-related metabolites	−0.007	(−0.015, 0.001)	0.081
Glucose	Glycolysis-related metabolites	−0.011	(−0.023, 0.002)	0.085
Alanine	Amino acids	−0.008	(−0.018, 0.003)	0.142
Glutamine	Amino acids	0.001	(−0.006, 0.007)	0.839
Histidine	Amino acids	−0.005	(−0.012, 0.002)	0.155
Glycine	Amino acids	0	(−0.015, 0.015)	0.965
Isoleucine	Amino acids	−0.007	(−0.02, 0.006)	0.277
Leucine	Amino acids	−0.006	(−0.017, 0.004)	0.251
Valine	Amino acids	0	(−0.008, 0.008)	0.994
Phenylalanine	Amino acids	−0.009	(−0.018, 0)	0.042 *
Tyrosine	Amino acids	−0.004	(−0.013, 0.004)	0.308
Acetate	Ketone bodies	−0.016	(−0.046, 0.014)	0.285

Acetoacetate	Ketone bodies	−0.032	(−0.065, 0.002)	0.067
3-Hydroxybutyrate	Ketone bodies	−0.023	(−0.053, 0.006)	0.124
Acetone	Ketone bodies	−0.009	(−0.022, 0.003)	0.146
Pyruvate	Ketone bodies	−0.015	(−0.031, 0)	0.057
Albumin	Fluid balance	0.002	(−0.002, 0.006)	0.407
Creatinine	Fluid balance	−0.012	(−0.022, −0.003)	0.014 *
Glycoprotein Acetyls	Inflammation	−0.007	(−0.014, −0.001)	0.02 *

* The metabolites that was related to healthy lifestyle. Significance, $p < 0.05$. [1] Multivariate linear regression analysis was conducted to adjust for age(continuous), sex(male/female), race (white or other), BMI (<25 kg/m^2, 25 to 29.9 kg/m^2, ≥30 kg/m^2), Townsend Deprivation Index(continuous), diabetes(yes/no), hypertension(yes/no) and family history of cardiovascular disease(yes/no)

Supplementary Table S5 Linear regression of metabolites and healthy lifestyle in cohort study[1]

Metabolites	Super Class	Coef.	95% CI	*p*
Concentration of Chylomicrons and Extremely Large VLDL Particles	Lipoprotein particle concentrations	−0.022	(−0.026, −0.019)	<0.001 *
Concentration of Very Large VLDL Particles	Lipoprotein particle concentrations	−0.012	(−0.015, −0.01)	<0.001 *
Concentration of Large VLDL Particles	Lipoprotein particle concentrations	−0.008	(−0.01, −0.006)	<0.001 *
Concentration of Medium VLDL Particles	Lipoprotein particle concentrations	−0.003	(−0.004, −0.002)	<0.001 *
Concentration of Small VLDL Particles	Lipoprotein particle concentrations	−0.003	(−0.004, −0.002)	<0.001 *
Concentration of Very Small VLDL Particles	Lipoprotein particle concentrations	−0.003	(−0.003, −0.002)	<0.001 *
Concentration of IDL Particles	Lipoprotein particle concentrations	−0.003	(−0.003, −0.002)	<0.001 *
Concentration of Large LDL Particles	Lipoprotein particle concentrations	−0.001	(−0.001, 0)	0.12
Concentration of Medium LDL Particles	Esterified cholesterol concentrations	−0.002	(−0.003, −0.002)	<0.001 *
Concentration of Small LDL Particles	Lipoprotein particle concentrations	−0.001	(−0.002, 0)	0.001
Concentration of Very Large HDL Particles	Lipoprotein particle concentrations	0.004	(0.003, 0.005)	<0.001 *
Concentration of Large HDL Particles	Lipoprotein particle concentrations	0.001	(0, 0.003)	0.116
Concentration of Medium HDL Particles	Lipoprotein particle concentrations	−0.006	(−0.007, −0.006)	<0.001 *
Concentration of Small HDL Particles	Lipoprotein particle concentrations	−0.005	(−0.006, −0.005)	<0.001 *
Concentration of VLDL Particles	Lipoprotein particle concentrations	−0.004	(−0.005, −0.0030	<0.001 *
Concentration of LDL Particles	Lipoprotein particle concentrations	−0.001	(−0.002, 0)	0.003
Concentration of HDL Particles	Lipoprotein particle concentrations	−0.005	(−0.005, −0.004)	<0.001 *
Total Lipids in Chylomicrons and Extremely Large VLDL	Lipoprotein particle concentrations	−0.021	(−0.024, −0.018)	<0.001 *
Total Lipids in Very Large VLDL	Total lipid concentrations	−0.012	(−0.014, −0.01)	<0.001 *
Total Lipids in Large VLDL	Total lipid concentrations	−0.007	(−0.009, −0.006)	<0.001 *
Total Lipids in Medium VLDL	Total lipid concentrations	−0.004	(−0.005, −0.003)	<0.001 *
Total Lipids in Small VLDL	Total lipid concentrations	−0.003	(−0.004, −0.002)	<0.001 *
Total Lipids in Very Small VLDL	Total lipid concentrations	−0.003	(−0.003, −0.002)	<0.001 *
Total Lipids in IDL	Total lipid concentrations	−0.003	(−0.004, −0.002)	<0.001 *
Total Lipids in Large LDL	Total lipid concentrations	−0.003	(−0.003, −0.002)	<0.001 *
Total Lipids in Medium LDL	Total lipid concentrations	−0.004	(−0.005, −0.003)	<0.001 *
Total Lipids in Small LDL	Total lipid concentrations	−0.002	(−0.002, −0.001)	<0.001 *
Total Lipids in Very Large HDL	Total lipid concentrations	0.007	(0.005, 0.008)	<0.001 *
Total Lipids in Large HDL	Total lipid concentrations	0.001	(0, 0.002)	0.194
Total Lipids in Medium HDL	Total lipid concentrations	−0.006	(−0.007, −0.006)	<0.001 *
Total Lipids in Small HDL	Total lipid concentrations	−0.006	(−0.006, −0.006)	<0.001 *
Total Lipids in VLDL	Total lipid concentrations	−0.006	(−0.007, −0.005)	<0.001 *
Total Lipids in LDL	Total lipid concentrations	−0.003	(−0.004, −0.002)	<0.001 *
Total Lipids in HDL	Total lipid concentrations	−0.004	(−0.004, −0.003)	<0.001 *
Phospholipids in Chylomicrons and Extremely Large VLDL	Total lipid concentrations	−0.025	(−0.03, −0.02)	<0.001 *
Phospholipids in Very Large VLDL	Phospholipid concentrations	−0.012	(−0.015, −0.009)	<0.001 *
Phospholipids in Large VLDL	Phospholipid concentrations	−0.011	(−0.014, −0.008)	<0.001 *
Phospholipids in Medium VLDL	Phospholipid concentrations	−0.003	(−0.004, −0.002)	<0.001 *
Phospholipids in Small VLDL	Phospholipid concentrations	−0.002	(−0.003, −0.002)	<0.001 *
Phospholipids in Very Small VLDL	Phospholipid concentrations	−0.002	(−0.003, −0.002)	<0.001 *
Phospholipids in IDL	Phospholipid concentrations	−0.002	(−0.003, −0.001)	<0.001 *
Phospholipids in Large LDL	Phospholipid concentrations	−0.003	(−0.004, −0.002)	<0.001 *
Phospholipids in Medium LDL	Phospholipid concentrations	−0.003	(−0.004, −0.003)	<0.001 *
Phospholipids in Small LDL	Phospholipid concentrations	0	(−0.001, 0.001)	0.885
Phospholipids in Very Large HDL	Phospholipid concentrations	0.009	(0.007, 0.011)	<0.001 *
Phospholipids in Large HDL	Phospholipid concentrations	−0.001	(−0.002, 0.001)	0.413
Phospholipids in Medium HDL	Phospholipid concentrations	−0.006	(−0.007, −0.006)	<0.001 *
Phospholipids in Small HDL	Phospholipid concentrations	−0.006	(−0.007, −0.006)	<0.001 *
Phospholipids in VLDL	Phospholipid concentrations	−0.006	(−0.007, −0.004)	<0.001 *
Phospholipids in LDL	Phospholipid concentrations	−0.003	(−0.003, −0.002)	<0.001 *

Phospholipids in HDL	Phospholipid concentrations	−0.004	(−0.005, −0.004)	<0.001 *
Cholesterol in Chylomicrons and Extremely Large VLDL	Phospholipid concentrations	−0.02	(−0.022, −0.017)	<0.001 *
Cholesterol in Very Large VLDL	Cholesterol concentrations	−0.008	(−0.01, −0.006)	<0.001 *
Cholesterol in Large VLDL	Cholesterol concentrations	−0.006	(−0.008, −0.004)	<0.001 *
Cholesterol in Medium VLDL	Cholesterol concentrations	−0.001	(−0.002, 0.001)	0.386
Cholesterol in Small VLDL	Cholesterol concentrations	−0.002	(−0.003, −0.001)	<0.001 *
Cholesterol in Very Small VLDL	Cholesterol concentrations	−0.002	(−0.003, −0.001)	<0.001 *
Cholesterol in IDL	Cholesterol concentrations	−0.003	(−0.004, −0.002)	<0.001 *
Cholesterol in Large LDL	Cholesterol concentrations	−0.002	(−0.003, −0.002)	<0.001 *
Cholesterol in Medium LDL	Cholesterol concentrations	−0.003	(−0.004, −0.002)	<0.001 *
Cholesterol in Small LDL	Cholesterol concentrations	−0.002	(−0.003, −0.001)	<0.001 *
Cholesterol in Very Large HDL	Cholesterol concentrations	0.006	(0.005, 0.007)	<0.001 *
Cholesterol in Large HDL	Cholesterol concentrations	0.003	(0.001, 0.005)	<0.001 *
Cholesterol in Medium HDL	Cholesterol concentrations	−0.006	(−0.006, −0.005)	<0.001 *
Cholesterol in Small HDL	Cholesterol concentrations	−0.005	(−0.005, −0.005)	<0.001 *
VLDL Cholesterol	Cholesterol concentrations	−0.004	(−0.005, −0.003)	<0.001 *
LDL Cholesterol	Cholesterol concentrations	−0.002	(−0.003, −0.002)	<0.001 *
HDL Cholesterol	Cholesterol concentrations	−0.003	(−0.003, −0.002)	<0.001 *
Cholesteryl Esters in Chylomicrons and Extremely Large VLDL	Cholesterol concentrations	−0.021	(−0.024, −0.018)	<0.001 *
Cholesteryl Esters in Very Large VLDL	Esterified cholesterol concentrations	−0.007	(−0.009, −0.005)	<0.001 *
Cholesteryl Esters in Large VLDL	Esterified cholesterol concentrations	−0.004	(−0.006, −0.002)	<0.001 *
Cholesteryl Esters in Medium VLDL	Esterified cholesterol concentrations	0.002	(0, 0.003)	0.076
Cholesteryl Esters in Small VLDL	Esterified cholesterol concentrations	−0.003	(−0.004, −0.002)	<0.001 *
Cholesteryl Esters in Very Small VLDL	Esterified cholesterol concentrations	−0.002	(−0.003, −0.001)	<0.001 *
Cholesteryl Esters in IDL	Esterified cholesterol concentrations	−0.003	(−0.004, −0.002)	<0.001 *
Cholesteryl Esters in Large LDL	Esterified cholesterol concentrations	−0.002	(−0.003, −0.002)	<0.001 *
Cholesteryl Esters in Medium LDL	Esterified cholesterol concentrations	−0.004	(−0.005, −0.003)	<0.001 *
Cholesteryl Esters in Small LDL	Esterified cholesterol concentrations	−0.003	(−0.004, −0.002)	<0.001 *
Cholesteryl Esters in Very Large HDL	Esterified cholesterol concentrations	0.006	(0.005, 0.008)	<0.001 *
Cholesteryl Esters in Large HDL	Esterified cholesterol concentrations	0.003	(0.002, 0.005)	<0.001 *
Cholesteryl Esters in Medium HDL	Esterified cholesterol concentrations	−0.005	(−0.006, −0.005)	<0.001 *
Cholesteryl Esters in Small HDL	Esterified cholesterol concentrations	−0.005	(−0.006, −0.005)	<0.001 *
Cholesteryl Esters in VLDL	Esterified cholesterol concentrations	−0.003	(−0.004, −0.002)	<0.001 *
Cholesteryl Esters in LDL	Esterified cholesterol concentrations	−0.003	(−0.004, −0.002)	<0.001 *
Cholesteryl Esters in HDL	Esterified cholesterol concentrations	−0.003	(−0.003, −0.002)	<0.001 *
Free Cholesterol in Chylomicrons and Extremely Large VLDL	Free cholesterol concentrations	−0.018	(−0.02, −0.015)	<0.001 *
Free Cholesterol in Very Large VLDL	Free cholesterol concentrations	−0.009	(−0.012, −0.007)	<0.001 *
Free Cholesterol in Large VLDL	Free cholesterol concentrations	−0.008	(−0.01, −0.006)	<0.001 *
Free Cholesterol in Medium VLDL	Free cholesterol concentrations	−0.002	(−0.003, −0.001)	<0.001 *
Free Cholesterol in Small VLDL	Free cholesterol concentrations	−0.002	(−0.003, −0.001)	<0.001 *
Free Cholesterol in Very Small VLDL	Free cholesterol concentrations	−0.002	(−0.003, −0.001)	<0.001 *
Free Cholesterol in IDL	Free cholesterol concentrations	−0.002	(−0.002, −0.001)	<0.001 *
Free Cholesterol in Large LDL	Free cholesterol concentrations	−0.002	(−0.003, −0.001)	<0.001 *
Free Cholesterol in Medium LDL	Free cholesterol concentrations	−0.002	(−0.003, −0.001)	0.002
Free Cholesterol in Small LDL	Free cholesterol concentrations	0.001	(0, 0.002)	0.005
Free Cholesterol in Very Large HDL	Free cholesterol concentrations	0.005	(0.005, 0.006)	<0.001 *
Free Cholesterol in Large HDL	Free cholesterol concentrations	0.002	(0, 0.003)	0.035
Free Cholesterol in Medium HDL	Free cholesterol concentrations	−0.006	(−0.007, −0.006)	<0.001 *
Free Cholesterol in Small HDL	Free cholesterol concentrations	−0.005	(−0.005, −0.005)	<0.001 *
Free Cholesterol in VLDL	Free cholesterol concentrations	−0.005	(−0.006, −0.004)	<0.001 *
Free Cholesterol in LDL	Free cholesterol concentrations	−0.001	(−0.002, 0)	0.002
Free Cholesterol in HDL	Free cholesterol concentrations	−0.003	(−0.004, −0.002)	<0.001 *
Triglycerides in Chylomicrons and Extremely Large VLDL	Triglyceride concentrations	−0.02	(−0.025, −0.014)	<0.001 *

Triglycerides in Very Large VLDL	Triglyceride concentrations	−0.013	(−0.016, −0.01)	<0.001 *
Triglycerides in Large VLDL	Triglyceride concentrations	−0.007	(−0.009, −0.005)	<0.001 *
Triglycerides in Medium VLDL	Triglyceride concentrations	−0.005	(−0.006, −0.004)	<0.001 *
Triglycerides in Small VLDL	Triglyceride concentrations	−0.004	(−0.005, −0.003)	<0.001 *
Triglycerides in Very Small VLDL	Triglyceride concentrations	−0.005	(−0.005, −0.004)	<0.001 *
Triglycerides in IDL	Triglyceride concentrations	−0.005	(−0.006, −0.004)	<0.001 *
Triglycerides in Large LDL	Triglyceride concentrations	−0.006	(−0.007, −0.005)	<0.001 *
Triglycerides in Medium LDL	Triglyceride concentrations	−0.006	(−0.007, −0.005)	<0.001 *
Triglycerides in Small LDL	Triglyceride concentrations	−0.006	(−0.007, −0.005)	<0.001 *
Triglycerides in Very Large HDL	Triglyceride concentrations	−0.004	(−0.005, −0.003)	<0.001 *
Triglycerides in Large HDL	Triglyceride concentrations	−0.007	(−0.008, −0.006)	<0.001 *
Triglycerides in Medium HDL	Triglyceride concentrations	−0.011	(−0.012, −0.01)	<0.001 *
Triglycerides in Small HDL	Triglyceride concentrations	−0.009	(−0.01, −0.008)	<0.001 *
Triglycerides in VLDL	Triglyceride concentrations	−0.008	(−0.01, −0.007)	<0.001 *
Triglycerides in LDL	Triglyceride concentrations	−0.006	(−0.007, −0.005)	<0.001 *
Triglycerides in HDL	Triglyceride concentrations	−0.009	(−0.01, −0.008)	<0.001 *
Apolipoprotein A1	Apolipoproteins	−0.004	(−0.005, −0.004)	<0.001 *
Apolipoprotein B	Apolipoproteins	−0.002	(−0.002, −0.001)	<0.001 *
Apolipoprotein B to Apolipoprotein A1 ratio	Apolipoproteins	0.003	(0.002, 0.003)	<0.001 *
Polyunsaturated Fatty Acids	Fatty acids	−0.001	(−0.001, 0)	0.002
Monounsaturated Fatty Acids	Fatty acids	−0.009	(−0.01, −0.009)	<0.001 *
Saturated Fatty Acids	Fatty acids	−0.008	(−0.009, −0.008)	<0.001 *
Docosahexaenoic Acid	Fatty acids	0.012	(0.011, 0.013)	<0.001 *
Linoleic Acid	Fatty acids	−0.002	(−0.002, −0.001)	<0.001 *
Omega-3 Fatty Acids	Fatty acids	0.012	(0.011, 0.013)	<0.001 *
Omega-6 Fatty Acids	Fatty acids	−0.002	(−0.003, −0.002)	<0.001 *
Total Fatty Acids	Fatty acids	−0.005	(−0.006, −0.005)	<0.001 *
Polyunsaturated Fatty Acids to Total Fatty Acids percentage	Fatty acids	0.005	(0.004, 0.005)	<0.001 *
Monounsaturated Fatty Acids to Total Fatty Acids percentage	Fatty acids	−0.004	(−0.004, −0.004)	<0.001 *
Saturated Fatty Acids to Total Fatty Acids percentage	Fatty acids	−0.003	(−0.003, −0.003)	<0.001 *
Docosahexaenoic Acid to Total Fatty Acids percentage	Fatty acids	0.018	(0.017, 0.019)	<0.001 *
Linoleic Acid to Total Fatty Acids percentage	Fatty acids	0.004	(0.004, 0.004)	<0.001 *
Omega-3 Fatty Acids to Total Fatty Acids percentage	Fatty acids	0.017	(0.016, 0.018)	<0.001 *
Omega-6 Fatty Acids to Total Fatty Acids percentage	Fatty acids	0.003	(0.003, 0.003)	<0.001 *
Total Cholines	Other lipids	−0.004	(−0.004, −0.004)	<0.001 *
Phosphatidylcholines	Other lipids	−0.004	(−0.005, −0.004)	<0.001 *
Sphingomyelins	Other lipids	−0.003	(−0.004, −0.003)	<0.001 *
Phosphoglycerides	Other lipids	−0.005	(−0.005, −0.004)	<0.001 *
Lactate	Glycolysis-related metabolites	−0.003	(−0.004, −0.002)	<0.001 *
Citrate	Glycolysis-related metabolites	0	(0, 0.001)	0.431
Glucose	Glycolysis-related metabolites	−0.002	(−0.003, −0.002)	<0.001 *
Alanine	Amino acids	0.002	(0.002, 0.003)	<0.001 *
Glutamine	Amino acids	0.002	(0.002, 0.003)	<0.001 *
Histidine	Amino acids	0.002	(0.001, 0.002)	<0.001 *
Glycine	Amino acids	0.005	(0.004, 0.006)	<0.001 *
Isoleucine	Amino acids	0	(−0.001, 0.001)	0.479
Leucine	Amino acids	0	(−0.001, 0.001)	0.528
Valine	Amino acids	0.001	(0.001, 0.002)	<0.001 *
Phenylalanine	Amino acids	−0.001	(−0.001, 0)	0.042
Tyrosine	Amino acids	0	(−0.001, 0)	0.144
Acetate	Ketone bodies	0.006	(0.004, 0.008)	<0.001 *
Acetoacetate	Ketone bodies	−0.014	(−0.017, −0.012)	<0.001 *
3-Hydroxybutyrate	Ketone bodies	−0.013	(−0.016, −0.011)	<0.001 *

Acetone	Ketone bodies	−0.004	(−0.005, −0.003)	<0.001 *
Pyruvate	Ketone bodies	−0.004	(−0.005, −0.003)	<0.001 *
Albumin	Fluid balance	0.001	(0.001, 0.001)	<0.001 *
Creatinine	Fluid balance	−0.002	(−0.002, −0.001)	<0.001 *
Glycoprotein Acetyls	Inflammation	−0.004	(−0.005, −0.004)	<0.001 *

* The metabolites that was related to healthy lifestyle. We performed a Bonferroni correction to correct the p value. Significance, $p < (0.05/161)$.

[1] Multivariate linear regression analysis was conducted to adjust for age (continuous), sex (male/female), race (white or other), BMI (<25 kg/m^2, 25 to 29.9 kg/m^2, ≥30 kg/m^2), Townsend Deprivation Index (continuous), diabetes (yes/no), hypertension (yes/no) and family history of cardiovascular disease(yes/no).

Supplementary Table S6 The association of healthy lifestyle-related metabolites with individual components of healthy lifestyle[1]

Metabolite	Coef. (SD)			
	Smoking Status	Alcohol Intake	Diet	Physical Activity
Docosahexaenoic Acid	0.0144 (0.00194) *	0.0236 (0.000973) *	0.0295 (0.000673) *	0.0260 (0.00303) *
Omega-3 Fatty Acids	0.00770 (0.00238) *	0.0223 (0.00119) *	0.0308 (0.000827) *	0.00902 (0.00372) *
Polyunsaturated Fatty Acids to Total Fatty Acids percentage	0.0164 (0.00494) *	−0.00111 (0.000250) *	0.00738 (0.000172) *	0.0127 (0.000774) *
Monounsaturated Fatty Acids to Total Fatty Acids percentage	−0.0153 (0.000589) *	−0.00286 (0.000297) *	−0.00720 (0.000205) *	−0.0156 (0.000920) *
Linoleic Acid to Total Fatty Acids percentage	0.0214 (0.000656) *	−0.00455 (0.000333) *	0.00194 (0.000230) *	0.0150 (0.001027) *
Omega-6 Fatty Acids to Total Fatty Acids percentage	0.0157 (0.000540) *	−0.00280 (0.000272) *	0.00337 (0.000189) *	0.0124 (0.000845) *
Creatinine	0.00579 (0.00972) *	−0.00251 (0.000489) *	−0.00613 (0.000339) *	−0.00622 (0.00151) *
Glycoprotein Acetyls	−0.0162 (0.000800) *	−0.00414 (0.000403) *	−0.00542 (0.000279) *	−0.0213 (0.00125) *

* Significance, $p < 0.001$.

All lifestyle factors were dichotomous and the unhealthy level was considered as the reference group. Metabolite concentrations were natural log10 transformed.

Supplementary Table S7 Performance of risk prediction models for incident heart failure [1]

Performance Metric	Models		*p* Value
	**Model I: Established Risk Factors ***	**Model II: Risk Factor Plus Metabolites #**	
C-**statistic (CI)**	0.788 (0.831, 0.851)	0.844 (0.834, 0.854)	<0.001
**Metrics of relative performance**			
IDI (CI)	Ref	0.002 (0.002, 0.004)	<0.001
Continuous NRI (CI)	Ref	0.048 (0.011, 0.077)	0.016

* The established risk factors included age, race, BMI, smoking status, alcohol status, physical activity, Townsend deprivation index, Diabetes and hypertension. # The combination model was the established risk factor plus the healthy lifestyle related-metabolites associated with heart failure, including m126 (docosahexaenoic acid), m128 (omega-3 fatty acids), m160 (creatinine). [1] The C-statistics was calculated to adjust for age (continuous), sex (male/female), race (white or other), BMI (<25 kg/m^2, 25 to 29.9 kg/m^2, ≥30 kg/m^2), Townsend Deprivation Index(continuous), smoking status (current/past/never), alcohol consumption (current/past/never)， physical exercise (continuous), diabetes (yes/no) and hypertension (yes/no).

Supplementary Table S8 Sensitivity Analysis[1]

Exclusion Criteria	C-Statistic (CI)	
	**Model I: Established Risk Factors ***	**Model II: Risk Factor Plus Metabolites #**
(1) Excluding the follow-up time <2 years	0.805 (0.795, 0.815)	0.843 (0.833, 0.853)
(2) Excluding the individuals with any cardiovascular disease at baseline (n=117016)	0.802 (0.792, 0.812)	0.842 (0.832, 0.852)

* The established risk factors included age, race, BMI, smoking status, alcohol status, physical activity, Townsend deprivation index, Diabetes and hypertension. # The combination model was the established risk factor plus the healthy lifestyle related-metabolites associated with heart failure, including m126 (docosahexaenoic acid), m128 (omega-3 fatty acids), m160 (creatinine). [1] The C-statistics was calculated to adjust for age (continuous), sex (male/female), race (white or other), BMI (<25 kg/m^2, 25 to 29.9 kg/m^2, ≥30 kg/m^2), Townsend Deprivation Index (continuous), smoking status (current/past/never), alcohol consumption (current/past/never)， physical exercise (continuous)， diabetes (yes/no) and hypertension (yes/no).
